# Human Cytomegalovirus IE2 Both Activates and Represses Initiation and Modulates Elongation in a Context-Dependent Manner

**DOI:** 10.1128/mbio.00337-22

**Published:** 2022-05-17

**Authors:** Christopher B. Ball, Ming Li, Mrutyunjaya Parida, Qiaolin Hu, Deniz Ince, Geoffrey S. Collins, Jeffery L. Meier, David H. Price

**Affiliations:** a Department of Biochemistry and Molecular Biology, University of Iowa, Iowa City, Iowa, USA; b Departments of Internal Medicine and Epidemiology, University of Iowa and Iowa City Veterans Affairs Health Care System, Iowa City, Iowa, USA; Princeton University

**Keywords:** human cytomegalovirus, IE2, DFF-ChIP, Pol II, preinitiation complex, transcription elongation, chromatin

## Abstract

Human cytomegalovirus (HCMV) immediate-early 2 (IE2) protein is a multifunctional transcription factor that is essential for lytic HCMV infection. IE2 functions as an activator of viral early genes, negatively regulates its own promoter, and is required for viral replication. The mechanisms by which IE2 executes these distinct functions are incompletely understood. Using PRO-Seq, which profiles nascent transcripts, and a recently developed DFF-chromatin immunoprecipitation (DFF-ChIP; employs chromatin digestion by the endonuclease DNA fragmentation factor prior to IP) approach that resolves occupancy and local chromatin environment, we show that IE2 controls viral gene transcription in three distinct capacities during late HCMV infection and reveal mechanisms that involve direct binding of IE2 to viral DNA. IE2 represses a subset of viral promoters by binding within their core promoter regions and blocking the assembly of preinitiation complexes (PICs). Remarkably, IE2 forms a repressive complex at the major immediate-early promoter region involving direct association of IE2 with nucleosomes and TBP. IE2 stimulates transcription by binding nearby, but not within, core promoter regions. In addition, IE2 functions as a direct roadblock to transcription elongation. At one locus, this function of IE2 appears to be important for the synthesis of a spliced viral RNA. Consistent with the minimal observed effects of IE2 depletion on host gene transcription, IE2 does not functionally engage the host genome. Our results reveal mechanisms of transcriptional control by IE2, uncover a previously unknown function of IE2 as a Pol II elongation modulator, and demonstrate that DFF-ChIP is a useful tool for probing transcription factor occupancy and interactions between transcription factors and nucleosomes at high resolution.

## INTRODUCTION

Human cytomegalovirus (HCMV) is a beta-herpesvirus that infects more than half of the world population and persists lifelong in its hosts. Although infection by HCMV is typically subclinical, compromised immunity enables unchecked viral replication and systemic infection that can cause life-threatening disease (1). Congenital HCMV infection currently represents the leading infectious cause of birth defects in the United States (2). Available antiviral drugs exhibit negative side effects and limited efficacy, and prolonged treatment has been associated with the development of drug-resistant HCMV variants (3). At present, there are no vaccines effective against HCMV.

The HCMV lytic infection cycle is characterized by a temporal cascade of gene expression facilitated by host polymerase II (Pol II) and its core transcriptional machinery. HCMV harbors an approximately 240-kb double-stranded DNA (dsDNA) genome and codes for hundreds of proteins and several noncoding RNAs (4, 5). A small immediate-early class of genes is the first to be expressed. This step is driven by recruitment of host transcription factors and Pol II to conserved *cis*-regulatory sequences on the HCMV genome at immediate-early promoter regions and does not require *de novo* protein synthesis (6). The immediate-early 1 and 2 (IE1 and IE2) proteins synergize with host factors to drive the expression of a kinetically distinct class of early genes whose products drive viral genome replication and the expression of viral late genes (7, 8). Three isoforms of IE2 are expressed during lytic infection, with apparent molecular masses of 86, 60, and 40 kDa. The IE2 p86 isoform, along with IE1, is expressed from the major immediate-early promoter (MIEP) immediately upon infection of cell types that yield viral progeny. Distinct mRNAs coding for IE1 and IE2 p86 arise through differential splicing. IE2 p86 is required for the expression of viral early genes and viral genome replication and negatively autoregulates its own promoter by engaging an element immediately upstream of the MIEP transcription start site (TSS) (9–11). Expression of IE2 p40 and IE2 p60 does not occur until late infection because these IE2 isoforms are driven by promoters that are recognized by a specialized type of Pol II preinitiation complex (PIC), consisting of viral late transcription factors (LTFs) that are preferentially recruited to promoters containing an upstream TATT element (12, 13). The three IE2 isoforms possess distinct N termini but share their C terminus and a core DNA-binding and dimerization domain (14, 15). The shared C terminus harbors transactivation domains that mediate interactions with TBP and TFIIB (16, 17) as well as a region that mediates interactions with the viral UL84 protein (18). IE2 also interacts with HDAC1 as well as the histone methyltransferases G9a and SUV39H1, which facilitate full MIEP repression, in a manner that depends on the C-terminal half of the IE2 protein (19). The extended N terminus of IE2 p86 is predicted to be largely unstructured but contains an additional transactivation domain that mediates interaction with TBP as well as a SUMO-interacting motif that facilitates interactions with the sumoylated TAF12 subunit of TFIID (16, 20).

Direct and indirect mechanisms of IE2 p86-mediated transactivation of viral early genes have been proposed. Existing models are nonmutually exclusive, reflect a significant number of documented IE2 protein-protein interactions, and have been informed in large part by reporter-type assays performed outside the context of a native HCMV infection. Models that do not invoke direct DNA binding by IE2 p86 suggest that it functions as a promiscuous transactivator through interactions with host transcription factors bound at their cognate sites or that it facilitates transcription initiation by engaging components of the PIC and behaving like a TATA-associated factor (20–22). On the other hand, direct engagement of viral DNA by IE2 has been associated with the activation of a subset of model viral early promoters, in conjunction with regulation by host transcription factors (23–26). Finally, IE2, in addition to IE1 and components of the viral tegument, have been suggested to counteract repressive chromatin on the HCMV genome and interfere with host intrinsic and innate immune responses, which fosters an environment conducive to viral gene expression and replication (27). Distinctly, IE2 p86 is well known to function in a negative feedback loop and repress its own promoter (MIEP) through engagement of a *cis*-repression sequence (*crs*) located between the initiator element and TATA box, thereby interfering with PIC assembly (9–11). This critical negative autoregulation is thought to promote productive infection by limiting the expression of cytotoxic IE2 p86 (28). The *crs* is a 14mer bounded by CG dinucleotides that contains a partially palindromic AT-rich core, consistent with IE2 engaging DNA as an inverted dimer. Indeed, IE2 exhibits specificity for the *crs* and *crs*-like sequences *in vitro* (29). Previous studies have also suggested that IE2 forms multimers or homo-oligomers *in vitro* and *in vivo*, which may impact IE2 function and DNA-binding properties (28, 29).

Levels of IE2 proteins accumulate through late infection (12). However, the roles of IE2 in transcriptional control during late infection remained elusive due to the challenges of generating replication-competent, IE2-deficient viruses (30, 31). Our recent efforts (12) overcame this obstacle by rapid depletion of an FKBP12(F36V) degron-tagged IE2 during late infection (32, 33). The transcriptional effects of IE2 depletion were assayed by PRO-Seq, which captures nascent transcripts and profiles Pol II transcription at single-nucleotide resolution (34). This study demonstrated that IE2 activates a specific subset of viral early-late and late promoters that tended to lack the upstream TATT core promoter element associated with LTF-driven transcription (12). Further investigation of these data for the purposes of the present study revealed a set of promoters other than the MIEP that are repressed by IE2 and evidence of a novel function of IE2 as a Pol II elongation barrier. To gain mechanistic insight into how IE2 differentially operates as an activator, repressor, and elongation barrier during late HCMV infection, standard IE2 chromatin immunoprecipitation sequencing (ChIP-Seq) and a novel method developed by our lab, DFF-ChIP, which employs chromatin digestion by the endonuclease DNA fragmentation factor prior to IP, were performed. Initial studies have demonstrated that DFF-ChIP significantly increases ChIP resolution and has the ability to inform on the local chromatin environment of immunoprecipitated factors through analysis of chromatin digestion patterns (35). Here, we show that IE2 engages numerous *crs*-like elements on viral chromatin during late infection and differentially influences transcription in a manner dependent on binding site context. Further, IE2 DFF-ChIP data reveal a functional association of IE2 with nucleosomes on viral chromatin, providing additional mechanistic insight into IE2 transcriptional control and demonstrating key aspects of DFF-ChIP that reflect its broader utility for the study of transcription factor-nucleosome interactions.

## RESULTS

### IE2 functions as a transcriptional activator, repressor, and elongation barrier.

**FIG 1:**
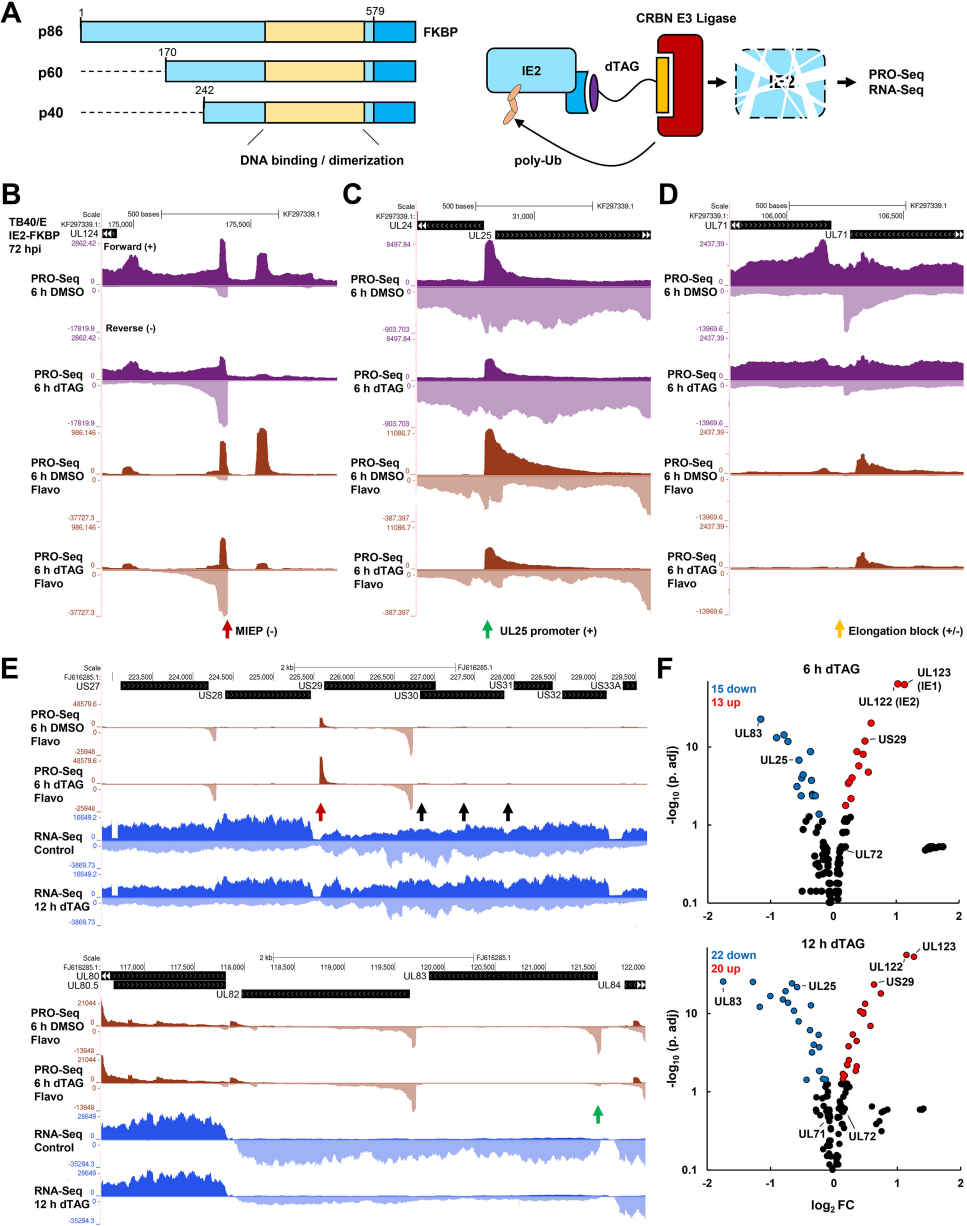
IE2 functions as a transcriptional repressor, activator, and elongation modulator. (A) All isoforms of IE2 are tagged at their C terminus with FKBP. Acute depletion of IE2 proteins is induced by treatment of cells with dTAG for the last 6 h of infection. HFF were infected with TB40/E IE2-FKBP for 72 h and with Towne IE2-FKBP for 96 h. Orthogonal readouts of nascent transcription and mRNA levels are carried out by PRO-Seq and RNA-Seq assays, respectively. (B) Genome browser tracks revealing derepression of the MIEP following IE2 depletion. Purple-colored PRO-Seq tracks always refer to data sets deriving from cells that were not treated with the P-TEFb inhibitor flavopiridol. Brown-colored PRO-Seq tracks always refer to data sets deriving from cells that were treated with flavopiridol to block pause-release during the last hour of infection. (C) Genome browser tracks showing a reduction in transcription of the UL25 gene following IE2 depletion. (D) Genome browser tracks showing a bidirectional pileup of PRO-Seq signal corresponding evidently to productively elongating Pol II between the convergently transcribed UL71 and UL72 genes. (E) PRO-Seq and RNA-Seq data are for Towne IE2-FKBP infections of HFF for 96 h. (Top) Genome browser tracks showing derepression of the US29 promoter by IE2 depletion and a corresponding increase in RNA-Seq coverage of the US29-US32 gene bloc. Black arrows indicate downstream promoter regions. (Bottom) Genome browser tracks showing a sharp reduction in transcription from the UL83 promoter following IE2 depletion and a corresponding decrease in the UL83-UL82 bicistronic message. (F) Volcano plots depicting significantly differentially expressed viral genes following 6 h (top) or 12 h (bottom) of IE2 depletion in HFF infected with Towne IE2-FKBP for 96 h. Significantly up- and downregulated genes (*P* adj. < 0.05) are colored, and discussed genes are indicated.

To better understand how loss of IE2 impacts host and viral gene expression during late HCMV infection, we further analyzed PRO-Seq data collected from primary human foreskin fibroblasts (HFF) infected by TB40/E or Towne strains of HCMV carrying FKBP12^F36V^-tagged IE2 and treated for the last 6 h of infection with dimethyl sulfoxide (DMSO) or dTAG (12). Addition of the degron domain to the IE2 C terminus was shown not to impact viral growth kinetics, and short-term depletion of IE2-FKBP during late infection did not affect viral DNA replication or the production of viral progeny. Furthermore, dTAG treatment had no effect on the abundance of untagged IE2 proteins (12). Rapid depletion of IE2 enables an assessment of the direct effects of IE2 on transcription. PRO-Seq data sets from cells additionally treated with flavopiridol (Flavo) for the last hour of infection were also analyzed. Flavo is a P-TEFb inhibitor that blocks release of paused Pol II into productive elongation (36). The amount of paused Pol II observed in the presence of Flavo is heavily dependent on initiation and is not dependent on entry into productive elongation, and it therefore represents a robust reporter of Pol II initiation. All PRO-Seq data sets were spike-in normalized and yielded reproducible effects across biological replicates (12). In addition, for the present study, total transcriptome sequencing (RNA-Seq) data sets were generated following 6- or 12-h treatments of HFF infected with a Towne strain of IE2-FKBP virus with dTAG to induce IE2 depletion. A schematic of this experimental framework is depicted in Fig. 1A. Western blot analysis of IE2-FKBP protein levels demonstrated that IE2 proteins were reduced to not more than approximately 20% of control levels by 12 h of dTAG treatment (see Fig. S1A in the supplemental material).

10.1128/mbio.00337-22.2 FIG S1 Depletion of Towne IE2-FKBP proteins for RNA-Seq and conservation of the elongation barrier effect in Towne virus. (A, top) Western blot analysis of IE2 proteins in HFF infected with Towne IE2-FKBP virus for 96 h and treated with DMSO for the last 6 h of infection or dTAG for the last 6 or 12 h of infection. (Bottom) Quantification of FKBP-tagged IE2 proteins at the indicated time points relative to the β-actin loading control. (B) Conservation of the elongation barrier effect in Towne virus. DMSO and dTAG treatments of HFF infected for 96 h with wild Towne virus indicate that diminishment of the elongation barrier effect is specific to IE2 depletion and not a result of dTAG treatment. Download FIG S1, PDF file, 0.4 MB. Copyright © 2022 Ball et al. 2022 Ball et al. https://creativecommons.org/licenses/by/4.0/ This content is distributed under the terms of the Creative Commons Attribution 4.0 International license.

Representative examples of the distinct transcriptional effects of IE2 depletion observed in PRO-Seq data with or without dTAG treatment and with or without Flavo are shown in Fig. 1B to D. As expected, IE2 depletion resulted in a substantial increase in paused Pol II transcripts at the MIEP, which drives the expression of IE1 and IE2 (encoded by UL123 and UL122, respectively) (Fig. 1B). A subset of viral promoters was activated by IE2, as exemplified by reductions in paused and productively elongating Pol II at the UL25 gene following IE2 depletion (Fig. 1C). A third and novel effect of IE2 as an apparent Pol II elongation modulator was observed, as an example, between the convergent UL71 and UL72 genes (Fig. 1D). This was signified by a bidirectional pileup of PRO-Seq signal adjacent to a site intervening the UL71 and UL72 coding sequences that was diminished following IE2 depletion. Equivalently scaled control and Flavo PRO-Seq data revealed that PRO-Seq signals across this region are primarily attributed to productively elongating Pol II, as the Flavo data revealed scant evidence of strong promoter activity. This apparent function of IE2 was conserved in the Towne strain of HCMV, for which additional controls, including dTAG treatment of cells infected with wild-type IE2 virus, revealed that the barrier effect was directly associated with IE2 depletion and not an indirect effect of dTAG treatment (Fig. S1B).

We compared PRO-Seq data analyses to changes in stable HCMV RNAs following IE2 depletion as measured by total RNA-Seq. Direct effects of IE2 depletion on transcription mirrored changes in abundance of viral mRNAs (Data Set S1). For example, IE2 depletion derepressed a strong promoter controlling transcription of the US29 gene, and this correlated with an increase in RNA-Seq coverage across a bloc of genes spanning US29 to US32 (Fig. 1E, top, red arrow). Although US30 to -32 are driven by unique upstream promoters (black arrows) (4), these data demonstrate that IE2-mediated repression of one promoter may impact downstream gene expression in cases where polycistronic transcripts are generated. Similarly, loss of IE2 is associated with a massive reduction in transcription from the UL83 promoter, and this was mirrored by a nearly 4-fold reduction in RNA-Seq coverage across the UL83 and UL82 genes within 12 h of dTAG treatment (Fig. 1E, bottom, green arrow). However, in this case, UL82 is associated with a comparably active core promoter region, and its translation is known not to be driven by the UL83-UL82 bicistronic message (12). Volcano plot representations of differentially expressed genes following 6 or 12 h of dTAG treatment are shown in Fig. 1F, with data points for discussed genes indicated. The UL71 and UL72 genes were not differentially expressed following IE2 depletion, indicating that the elongation barrier function of IE2 does not critically regulate the expression of these mRNAs. However, it is possible that effects are masked by the high stability of mRNAs and short-term depletion of IE2 in these experiments. More detailed investigations of the potential importance of this IE2 function are presented later. Overall, after 12 h of IE2 depletion, 42/155 annotated and measured HCMV transcripts exhibited significant differential expression (adjusted *P* value [*P* adj.] of <0.05) (Data Set S1). In contrast, only 118/8,132 detectably expressed host genes exhibited significant changes in expression, all of which were small (<2-fold; Data Set S1). Consistent with this, depletion of IE2 shows virtually no effects on host gene transcription by PRO-Seq (12).

10.1128/mbio.00337-22.1 Data Set S1 (1) Nucleic acid reagents utilized in the study; (2) results of RNA-Seq differential gene expression analysis by DESeq2 for viral and host genes; (3) a summary of peaks and motifs called for IE2 ChIP datasets; (4) a table documenting the distance between all maximum TSSs on the viral genome, their dependence on IE2 as measured by PRO-Seq, and their distance to the nearest IE2 binding site; (5) a list of TSS to IE2 binding site distances used for the analysis in Fig. 5B. Download Data Set S1, XLSX file, 3.5 MB. Copyright © 2022 Ball et al. 2022 Ball et al. https://creativecommons.org/licenses/by/4.0/ This content is distributed under the terms of the Creative Commons Attribution 4.0 International license.

### IE2 preferentially engages a consensus binding site on viral chromatin during late infection.

**FIG 2:**
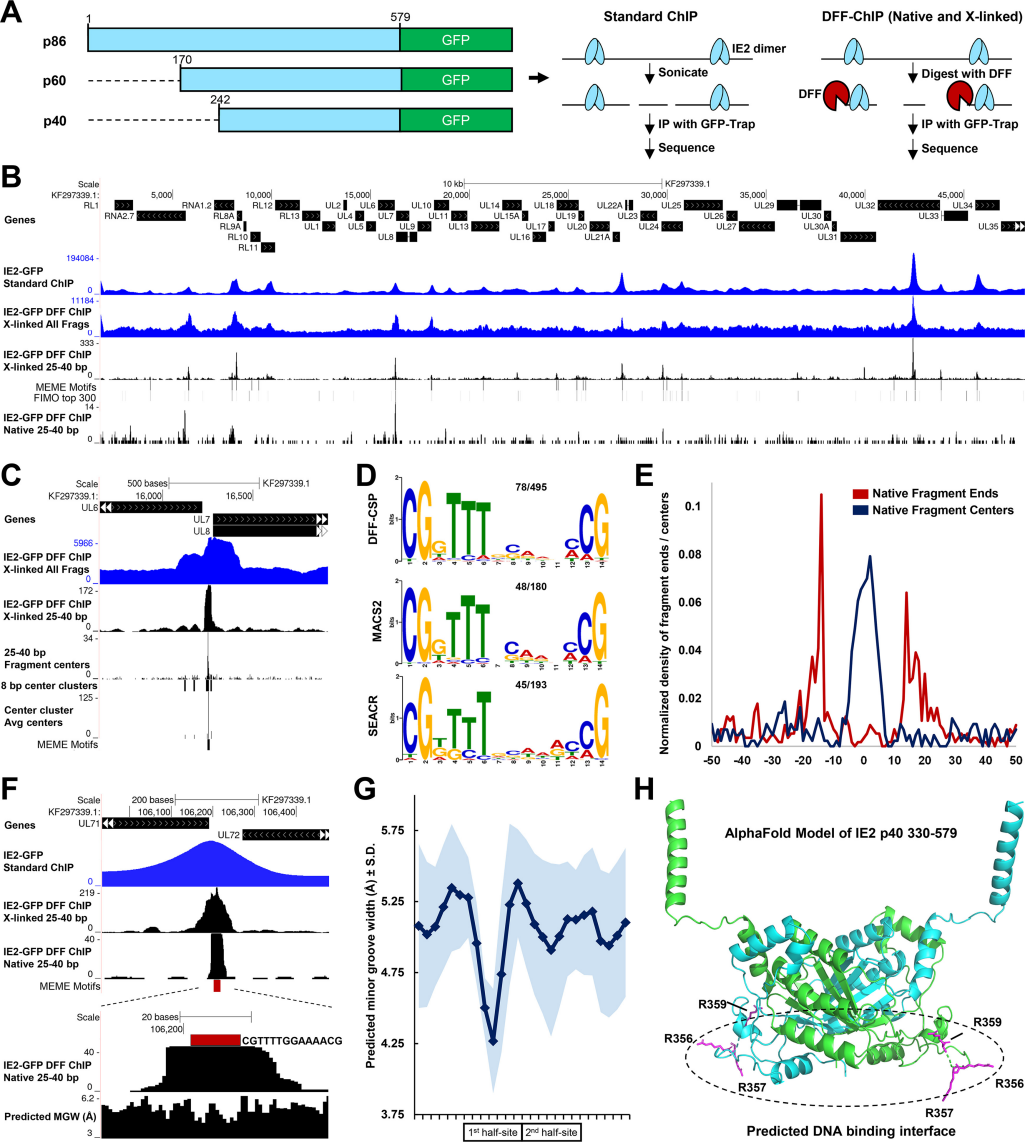
IE2 preferentially engages a consensus motif on the viral genome during late infection. (A) Schematic depicting GFP-tagged isoforms of IE2 as well as standard ChIP-Seq and native or cross-linked DFF-ChIP assays utilized to assay IE2 occupancy on the host and viral genomes. (B) Broad view of the HCMV genome showing IE2-GFP ChIP signals as detected at 48 hpi by standard ChIP-Seq, non-size-selected IE2-GFP DFF-ChIP, and 25- to 40-bp fragments deriving from cross-linked and native IE2-GFP ChIP data sets. Tracks corresponding MEME motifs defined as is shown in panels C and D and FIMO motifs described in the text are shown. (C) Depiction of the steps of DFF-CSP, showing the raw DFF-ChIP data, 25- to 40-bp fragment size-selected DFF-ChIP data, pileups of 25- to 40-bp fragment centers, identification of center clusters, determination of the average center position, and location of the relevant consensus IE2 binding site shown in panel D. (D) Results of MEME analyses performed on sequences derived from DFF-CSP, MACS2, and SEACR peak-called regions from 25- to 40-bp cross-linked IE2-GFP DFF-ChIP data. The top-scoring motif from each analysis is shown. The fraction of analyzed sequences containing motifs is indicated. (E) Metaplots of 25- to 40-bp fragment ends and centers derived from native IE2-GFP DFF-ChIP data, centered on 78 defined IE2 binding sites. (F) Zoomed-in view of a high-occupancy IE2 consensus site between the UL71 and UL72 genes, showing the improvement in resolution and signal-to-noise ratio of cross-linked and native DFF-ChIP compared to standard ChIP following size selection. (G) Plot of the average predicted minor groove width at DNA positions across and flanking 78 defined IE2 binding sites. Data are shown as averages ± standard deviations. (H) Top-ranked Colabfold structural model of an IE2 p40 dimer. The well-structured region between amino acids 330 and 579 is shown. Locations of arginine residues between aa 356 and 359 critical for DNA binding and possibly minor groove interaction are indicated within a predicted DNA binding interface.

Sequence gazing provided initial support for a model in which the transcriptional effects of IE2 depletion in late infection are mechanistically linked to IE2 DNA binding at *crs*-like elements. Consistent with this idea, certain model early promoters, including the RNA1.2 promoter and UL112/113 promoter, which are stimulated by IE2 p86 in reporter assays and contain upstream proximal *crs*-like elements (8, 24–26), were found to exhibit reduced levels of paused Pol II following IE2 depletion. As the detailed genome-wide occupancy of IE2 on host and viral chromatin had not been investigated, a TB40/E mCherry virus in which IE2 was C-terminally tagged with green fluorescent protein (GFP) was generated that enabled ChIP experiments to be carried out with high-affinity GFP nanobody technology (Fig. 2A and Fig. S2A and B; also see Materials and Methods). Fusion of GFP or yellow fluorescent protein (YFP) to the IE2 C terminus has previously been shown not to impair viral growth kinetics (28, 37). Both standard ChIP-Seq and DFF-ChIP for IE2-GFP were then performed with HFF infected for 48 h with TB40/E mCherry IE2-GFP. In our previous work, DFF-ChIP was carried out only under native conditions (35). Here, to preserve interactions between IE2 and chromatin, DFF-ChIP was also carried out on cross-linked cells.

10.1128/mbio.00337-22.3 FIG S2 Generation of TB40/E mCherry IE2-GFP, reproducibility of ChIP-Seq data, and further analysis of IE2 DNA binding properties. (A) Restriction fragment polymorphism analysis of wild type TB40/E BAC, a BAC containing a GalK cassette at the IE2 C terminus, and a BAC containing a GFP coding sequence inserted at the IE2 C terminus. Asterisks indicate fragments modified to insert the GalK cassette or GFP at the IE2 C terminus. (B) Live cell fluorescence microscopy image of an HFF transfected with TB40/E mCherry IE2-GFP BAC. IE2-GFP localizes to the nucleus as expected. (C) Pearson’s correlation analysis of analyzed ChIP-Seq data sets. (D) Broad genome browser view of IE2-GFP ChIP-Seq, previously published TB40/E 20- and 72-hpi IE2 ChIP-Seq, and previously published 72-hpi UL84 ChIP-Seq. The top MEME motif detected by analysis of sequences from MACS2 peaks called from standard IE2-GFP ChIP-Seq data is shown at the top right of the IE2-GFP ChIP tracks. Peaks of paused Pol II on the HCMV do not correlate with IE2 or UL84 datasets. (E) Broad genome browser view of IE2-GFP ChIP-Seq, 20- to 100-bp fragments from cross-linked IE2-GFP DFF-ChIP, and Pol II ChIP-Seq on the host genome. These data clearly demonstrate the high quality of Pol II ChIP-Seq data. Peaks in the IE2 ChIP-Seq data modestly correlate with sites of paused Pol II and largely do not overlap IE2 consensus matches predicted with FIMO. (F) Predicted IDDT values for amino acid positions in IE2 p40 for five ranked models. The approximate location of the DNA binding and dimerization domain is indicated. Higher IDDT values indicate greater confidence in the model. (G) Surface electrostatics for the IE2 p40 dimer (aa 330 to 579) oriented as shown in Fig. 2H (left) and rotated 90° to show the predicted DNA binding interface (right). Download FIG S2, PDF file, 1.4 MB. Copyright © 2022 Ball et al. 2022 Ball et al. https://creativecommons.org/licenses/by/4.0/ This content is distributed under the terms of the Creative Commons Attribution 4.0 International license.

Enrichment of IE2-GFP standard ChIP-Seq signals was observed over numerous sites on the viral genome (Fig. 2B). Furthermore, it was noted that a disproportionate ~30% of IE2 standard ChIP reads mapped to the viral genome despite there being much less viral DNA than host DNA (Data Set S1, mapping statistics). Pearson’s correlation analysis of standard IE2-GFP-ChIP duplicates indicated a high level of reproducibility (*r* = 0.95) (Fig. S2C). IE2-GFP DFF-ChIP data from cross-linked cells and 25- to 40-bp fragments deriving from IE2-GFP DFF-ChIP data from cross-linked and native cells are shown in the same window. Fragment size selection *in silico* was carried out in prior studies that coupled MNase digestion to ChIP (38, 39), with the logic that the smallest enriched fragments hone in on the site of factor occupancy. Clearly, 25- to 40-bp fragments from cross-linked IE2-GFP DFF-ChIP showed very sharp peaks and a significant increase in signal-to-noise ratio compared to standard ChIP. Peaks observed across data sets also exhibited a high level of visual correlation. However, few peaks were observed for the native IE2-GFP DFF-ChIP data, which is consistent with the idea that IE2, like most other transcription factors, transiently engages DNA. Of note, a recently published study documented ChIP-Seq for IE2 and its known interactor, UL84, in cells infected with wild-type TB40/E virus (40). Analyses in their study focused on occupancy at the MIEP and origin of lytic replication. We processed their raw data for comparison. Our IE2-GFP standard ChIP duplicates highly correlated with their IE2 ChIP at 72 h postinfection (hpi) (Pearson’s *r* = 0.76 to 0.81), and, very interestingly, correlated positively with UL84 ChIP at 72 hpi (*r* = 0.63 to 0.70) (Fig. S2C). Genome browser views of these data revealed many sites of apparent IE2 and UL84 cooccupancy (Fig. S2D). Although IE2 and UL84 stoichiometry cannot be inferred from these data, this question is of interest, as UL84 has previously been suggested to attenuate IE2-mediated transactivation and enhance repression (41). Our IE2-GFP ChIP correlated poorly with their IE2 ChIP performed at 20 hpi (*r* = 0.32 to 0.35) (Fig. S2C), which may reflect dynamic association of IE2 with viral chromatin between early and late stages of infection or technical challenges to ChIP posed by the low copy number of viral genomes prior to replication.

A novel peak-calling approach to determine sites of IE2 occupancy from IE2-GFP DFF-ChIP data was developed and dubbed DFF-ChIP-Seq peak (DFF-CSP) (see Materials and Methods for link to source code). The premise of its approach is illustrated for one site of IE2 occupancy at the UL7/8 promoter region in Fig. 2C. From non-size-selected DFF-ChIP data, a fragment size range of interest is chosen, in this case 25 to 40 bp. Individual fragment centers are determined, and a scanning window function is applied that identifies regions, termed center clusters, that pass a user-defined threshold level of fragment center density. Here, center clusters were defined as 8-bp windows containing at least ten 25- to 40-bp fragment centers. The average center position within each center cluster is then determined. Sequence ±20 bp relative to this single base pair position was queried for motif enrichment. We utilized DFF-CSP, in addition to other peak-calling algorithms, including MACS2 (42) and SEACR (43), to call peaks from 25- to 40-bp cross-linked IE2-GFP DFF-ChIP data. A total of 496, 180, and 193 peaks were detected by DFF-CSP, MACS2, and SEACR, respectively. MEME (44) analyses of sequence surrounding identified peaks in DFF-ChIP data uncovered 78, 48, and 45 unique consensus motifs from the aforementioned 495, 180, and 193 peaks, respectively, and converged uniformly on a 14-bp motif bounded by CG dinucleotides (Fig. 2D). This motif is similar to the previously characterized *crs* for which IE2 exhibits specificity *in vitro* (29). MEME analysis of sequence covering MACS2 peaks identified from standard ChIP identified a nearly identical consensus (Fig. S2D). A summary of peak calling and motif detection and location data is provided in Data Set S1, peaks and motifs summary. The core of the enriched motif was AT-rich, although T-richness within only one half-site was detected (Fig. 2D). A weak preference for a GC spacer between the two half-sites was also noted. The majority (65%) of 129 unique IE2 consensus motifs collectively detected by all approaches were found using DFF-CSP. Later analyses therefore focused on the 78 sites detected using this approach. FIMO (45) was utilized to investigate whether peaks in the IE2-GFP DFF-ChIP data not associated with a MEME motif contained similar sequences. Many of the top 300 matches to the IE2 consensus motif exhibited correlation with 25- to 40-bp cross-linked IE2-GFP DFF-ChIP peaks (Fig. 2B). Thus, we conclude that IE2 preferentially engages numerous *crs*-like motifs on the HCMV genome during late infection.

In contrast, analysis of IE2-GFP ChIP signals on the host genome did not indicate any obvious sites of enrichment, apart from a modest correlation with Pol II in promoter regions (Fig. S2E). FIMO was further utilized to identify sites on the host genome corresponding to the MEME motif identified from IE2 occupancy on the HCMV genome. No strong visual correlation between these sites and IE2-GFP ChIP data was evident (Fig. S2E). Host gene promoter occupancy by IE2 may be analogous to a recent report indicating that HSV-1 ICP4 associates with host promoters independent of its sequence preferences (46). However, this occupancy by IE2 is not functional, as IE2 depletion does not impact host gene transcription. Thus, we conclude that IE2 does not exhibit extensive or sequence-specific engagement of host chromatin during late infection, consistent with the minimal effects of its depletion on host gene transcription.

To demonstrate the high possible resolution of DFF-ChIP, metaplots of fragment ends and fragment centers at identified IE2 binding sites were generated for native 25- to 40-bp IE2-GFP DFF-ChIP fragments (Fig. 2E). It was noted that native IE2-GFP DFF-ChIP captured occupancy at a subset of IE2 binding sites (exemplified in Fig. 2F). Fragment ends defined a sharp, approximately 28-bp footprint of IE2, with fragment centers falling proximal to the binding site center (Fig. 2E). While cross-linking was necessary to capture IE2 binding at most sites, fragment ends and centers were clearly more heterogeneous for the cross-linked sample (Fig. 2F), possibly relating to adjacent protein-DNA interactions retained by cross-linking or cross-linking heterogeneity across cells. Interestingly, IE2 occupancy at the region between the UL71 and UL72 genes situated squarely on a nearly palindromic 14mer motif, CGTTTTGGAAAACG, suggesting that IE2 DNA binding is associated with its elongation barrier function.

Previous studies have strongly suggested that IE2 interacts primarily with the DNA minor groove (29, 47). The minor groove narrows in the context of T- or A-rich stretches that are at least three nucleotides in length, and this narrowing potentiates electrostatic interactions between basic residues in DNA-binding proteins and the DNA phosphate backbone (48). DNAPhi (49) was utilized to predict minor groove widths across the HCMV genome and at IE2 binding sites in aggregate. Dips in minor groove width were, as expected, apparent in both half-sites at the IE2 binding site between the UL71 and UL72 genes (Fig. 2F). Concordant with the defined IE2 consensus motif, a substantial decrease in the average minor groove width for all defined IE2 binding sites was observed in the T-rich half (Fig. 2G). IE2 contains a basic RRGR motif between residues 356 and 359 that is similar to motifs observed within minor groove-interacting HMG domains and is essential for DNA binding *in vitro* (29). As crystal structures of IE2 are yet lacking, Colabfold was utilized to generate a homology-based structural model of an IE2 p40 dimer (Fig. 2H) (50, 51). Most of the protein between positions 330 to 579, inclusive of the minimal DNA binding region, was modeled with high confidence (Fig. S2F). Interestingly, one surface of the dimer contains a cluster of positive charges that would facilitate interaction with DNA (Fig. S2G), and the RRGR motif is positioned within a flexible loop along this interface (Fig. 2H). Prediction of the DNA binding interface was also based on similarity to the HHV-6A IE2-CTD structure (52). Taken together, these data strongly suggest that IE2 engages numerous consensus motifs resembling the *crs* in late HCMV infection, that IE2 does not functionally engage the host genome, and that IE2 minor groove contacts are likely important for DNA binding *in vivo*. In addition, DFF-ChIP is presented here as a useful tool for characterizing transcription factor occupancy with improved resolution.

### IE2 can form a complex with nucleosomes on viral chromatin.

**FIG 3:**
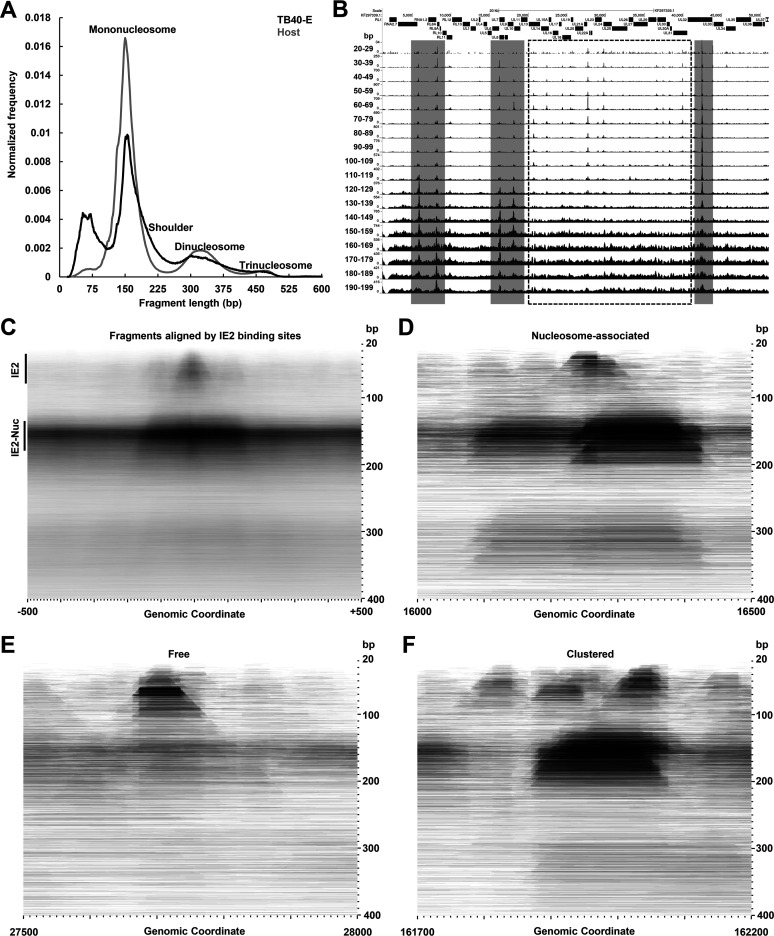
IE2 interacts with nucleosomes on viral chromatin. (A) Fragment size distribution plots of cross-linked IE2-GFP DFF-ChIP data on the viral and host genomes. Data were normalized to total signal and derived from a single library that was PCR amplified, mapped simultaneously to the host and viral genomes, and deduplicated, as described in Materials and Methods. Fragments corresponding to mono-, di-, and trinucleosomes are indicated, and a shoulder to the mononucleosome-sized peak on the viral genome is marked. (B) Genome browser view of cross-linked IE2-GFP DFF-ChIP tracks fragment size selected for 10-bp intervals spanning 20 to 200 bp. Gray-shaded regions contain peaks of IE2 occupancy that also exhibit peaks of larger fragments potentially corresponding to nucleosome enrichment, whereas peaks of IE2 occupancy within the black-dashed box largely do not show this enrichment. (C) FragMap of cross-linked IE2-GFP DFF-ChIP data representing all identified IE2 binding sites except for the *crs*. The FragMap is centered on IE2 binding sites and extends ±500 bp. Enriched signals corresponding to IE2 alone (IE2) and IE2-nucleosomes (IE2-Nuc) are indicated. (D, E, and F) FragMaps showing examples of nucleosome-associated, free, and clustered modes of IE2 DNA binding.

As DFF-ChIP has recently been shown to retain information about the local chromatin environment of immunoprecipitated factors, we explored whether IE2-GFP DFF-ChIP data bore insight regarding IE2 association with nucleosomes or other complexes on the viral genome. To this end, fragment size distribution plots of IE2-GFP DFF-ChIP data for the TB40/E and host genomes were generated (Fig. 3A). An enrichment of fragments <100 bp in size was clearly apparent on the HCMV genome that likely represents footprints of bound IE2. The major peaks over approximately 150 bp derive most likely from mononucleosomes. These signals represent an apparent background on both the host and viral genomes, which is unsurprising given the abundance of nucleosomes on the host genome and the resilience of nucleosomal DNA to DFF digestion (53). Notably, a shoulder extended from the mononucleosome peak on the TB40/E genome, which could represent IE2 bound adjacent to a nucleosome. Signals corresponding to di- and trinucleosome-sized fragments were present but less evident, reflecting the robust extent of DFF digestion.

Several studies support the conclusion that the viral genome is broadly accessible during lytic infection. HCMV enters and leaves cells with a genome denuded of chromatin (54). The HCMV genome is highly permissive to transcription initiation during late infection, and Pol II DFF-ChIP data strongly suggest that the transcriptional machinery does not interface extensively with chromatin (35, 55). ATAC-Seq data for HCMV in semipermissive Kasumi-3 cells indicate broad accessibility (56). MNase digestion studies suggest generally low but variable nucleosome occupancy across viral loci (54, 57). HCMV encodes factors that counteract repressive host chromatin from the onset of infection (27, 57, 58). There are approximately 100 viral genomes in late infected cells, adding an additional layer of complexity in that chromatin states across viral templates are likely different. However, several studies have reported on epigenetic control of viral gene expression during lytic infection, particularly at the MIEP (19, 59, 60). Remarkably, IE2-GFP DFF-ChIP appeared to enrich for nucleosomes adjacent to IE2 binding sites at some viral loci, as reflected by extended signals at IE2-bound sites in pileup tracks spanning fragments 20 to 200 bp in size (Fig. 3B, compare gray-shaded, possibly nucleosome-enriched sites to sites with no apparent nucleosome enrichment in the black dashed box). Protection of an approximately nucleosome-sized complex on either side of IE2 bound at the UL7/8 promoter region was also evident in non-size-selected DFF-ChIP data in Fig. 2C.

To further investigate a possible IE2-nucleosome interaction on viral chromatin, a fragMap for cross-linked IE2-GFP DFF-ChIP data was generated. A fragMap is a 2-dimensional heatmap of fragment position versus fragment size, described in Spector et al. (35). The fragMap represents all identified IE2 binding sites except for the *crs*, which was removed from the analysis due to its high IE2 occupancy (Fig. 3C). Small fragments were clearly enriched over sites directly bound by IE2, and interestingly, an enrichment of approximately nucleosome-sized fragments extended from either side of bound IE2. This result suggests that IE2 can associate immediately adjacent to a nucleosome or potentially within it. Properties of IE2 binding were further investigated genome-wide and in relation to recently published DFF-ChIP data for H3K4 trimethylated nucleosomes and TBP through the construction of 21-kb-interval fragMaps for each data set that span the entire viral genome (Fig. S3). For each individual data set, the black value was held constant for all the individual fragMaps, allowing for comparison of signals across the entire genome. The 78 consensus IE2 binding sites are marked. This view of IE2-GFP DFF-ChIP data further demonstrated that IE2 occupancy is enriched over consensus motifs and is extraordinarily high over the MIEP but also showed a low to moderate level of IE2 occupancy, reflected by discrete protection across the entire viral genome. Although nucleosome-sized complexes were detected across the viral genome, nucleosomes flanking consensus IE2 binding sites were preferentially enriched. This enrichment generally did not correlate with the abundance of H3K4me3 nucleosomes, although there is a notable congruency in nucleosome position between the IE2-GFP and H3K4me3 DFF-ChIP data sets. In addition, IE2 occupancy did not correlate well with DFF-ChIP data for TBP, which defines the position of Pol II PICs (35), an important observation in light of the fact that TBP and other components of TFIID are documented IE2 interactors (16, 21).

10.1128/mbio.00337-22.4 FIG S3 HCMV genome-spanning fragMaps for IE2-GFP, H3K4me3, and TBP. fragMaps (21 kb) for TBP, IE2-GFP, and H3K4me3 DFF-ChIP at 48 hpi representing the entire HCMV genome are shown. The 78 consensus IE2 binding sites are indicated as small black bars across the top of each IE2-GFP fragMap. Black values for each dataset (TBP, IE2-GFP, or H3K4me3) are set to the same value, enabling comparison of signals across fragMaps. Regions corresponding to RNA1.2, RNA2.7, RNA4.9, and MIEP promoter regions are indicated, as is the site of the UL71/72 elongation barrier. The RNA4.9 promoter region is located at the right side of a broader region designated *ori*Lyt. Download FIG S3, PDF file, 6.8 MB. Copyright © 2022 Ball et al. 2022 Ball et al. https://creativecommons.org/licenses/by/4.0/ This content is distributed under the terms of the Creative Commons Attribution 4.0 International license.

Study of fragMaps at individual IE2 binding sites indicated heterogeneity in the local chromatin environment of bound IE2. For example, IE2 bound at the repressed UL7/8 promoter region can be associated with a nucleosome on either side (Fig. 3D, nucleosome-associated), while IE2 bound upstream of the activated UL22A gene appears not to associate with flanking nucleosomes (Fig. 3E, free). Previous work identified three nearby sites upstream of the activated UL112 promoter that are bound by IE2 *in vitro* (25), and each appeared to be engaged *in vivo* (Fig. 3F, clustered). These results are consistent with the notion that the HCMV genome exists in an irregular chromatin state. Overall, our analyses suggest that IE2 broadly engages the HCMV genome but is guided by specific interactions with consensus motifs and can be associated with nucleosomes.

### IE2 establishes a unique repressed architecture at MIEP and acts as a repressor on many promoters by binding to core promoter regions.

**FIG 4:**
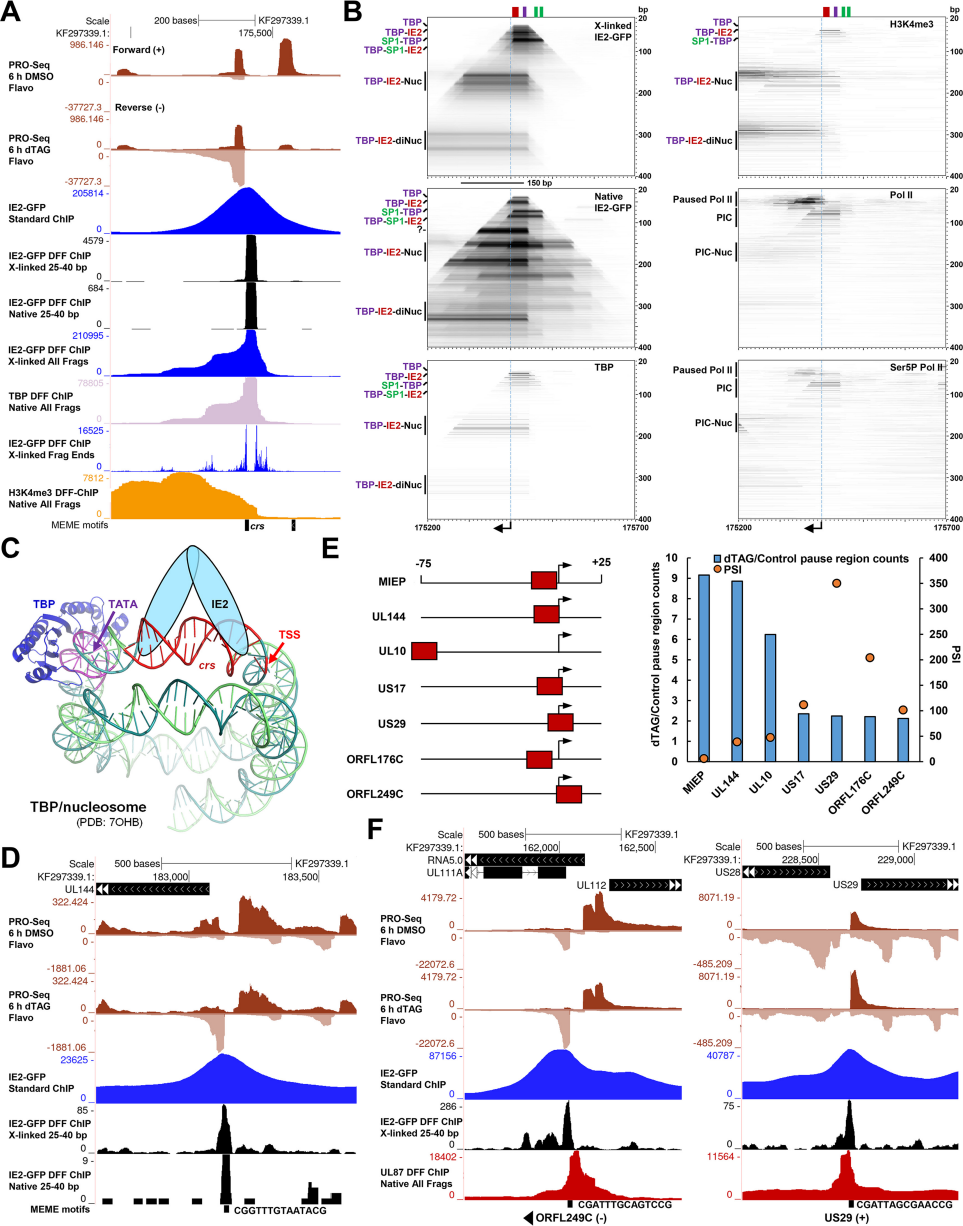
IE2 associates with repressive chromatin at the MIEP and represses transcription by inhibiting PIC assembly. (A) Genome browser view of PRO-Seq (TB40/E IE2-FKBP 72 hpi Flavo, with our without 6-h dTAG), standard IE2-GFP ChIP-Seq, cross-linked and native IE2-GFP DFF-ChIP (25 to 40 bp and all fragments), cross-linked IE2-GFP DFF-ChIP fragment ends from all fragments, and native H3K4me3 and TBP DFF-ChIP at the MIEP region. (B) FragMaps of cross-linked and native IE2-GFP DFF-ChIP and native TBP, H3K4me3, Pol II, and Ser5P Pol II DFF-ChIP data at the MIEP region. The positions of the major TSS, *crs*, TATA box, and SP1 sites in the proximal enhancer are indicated. Identifiable complexes reflected by different populations of enriched fragments are indicated at the left of each fragMap. (C) Structural model of the IE2-repressed MIEP containing TBP bound at the TATA box and IE2 bound at the downstream *crs* in the context of a nucleosome. Histones are not shown. The position of the TSS is indicated. (D) Genome browser tracks showing derepression of the UL144 promoter upon IE2 depletion and 25- to 40-bp IE2-GFP DFF-ChIP data revealing IE2 occupancy of a consensus motif within the UL144 core promoter region. (E, left) Graphical depiction of the locations of IE2 binding at all promoters derepressed at least 2-fold by IE2 depletion. (Right) Dual-axis plot of fold induction of promoters indicated at the left following IE2 depletion and PFA-sensitivity index of the promoter. (F) Genome browser tracks of the US29 putative ORFL249C promoter regions showing derepression of transcription by IE2 depletion, IE2 engagement of the core promoter region, and UL87 occupancy by native DFF-ChIP.

Negative autoregulation of the MIEP by IE2 is a well-established function that involves binding of IE2 to the *crs*, a consensus motif located between the major transcription start site (TSS) and TATA box (9–11). The MIEP exhibited the greatest level of IE2-GFP occupancy across all ChIP data sets, and 25- to 40-bp DFF-ChIP fragments presumably representing IE2 binding to the *crs* were abundant (Fig. 4A and Fig. S3). Interestingly, the non-size-selected IE2-GFP DFF-ChIP data sets contained signals consistent with up to 3 associated, close-packed nucleosomes downstream of the TSS, as evidenced by step-like extensions of approximately 150 bp (Fig. 4A). A track of fragment ends demarcated the prominent site of IE2 occupancy within the core promoter region and demonstrated digestion within the DNA separating some of the downstream nucleosomes. Native DFF-ChIP for H3K4me3 nucleosomes showed protuberances of signal that are approximately demarcated by IE2-GFP DFF-ChIP fragment ends, and the H3K4me3 signal shared a sharp upstream boundary with complexes protected by IE2, further supporting the conclusion that IE2 is engaged with nucleosomes at the MIEP (Fig. 4A).

Interestingly, it was noted that a major fraction of 25- to 40-bp IE2-GFP DFF-ChIP fragment ends dissected the *crs*. This would not be expected if the *crs* was fully protected from DFF by engaged IE2 (Fig. 4A). As this prompted further investigation of the mode of IE2 binding at the MIEP, we generated fragMaps for a 500-bp region spanning the MIEP for cross-linked and native IE2-GFP DFF-ChIP and for recently reported TBP, H3K4me3, Pol II, and Ser5P Pol II DFF-ChIP data sets (Fig. 4B) (35). The position and orientation of the MIEP major TSS is indicated, and the locations of the *crs* (red), TATA box (purple), and upstream SP1/SP3 sites (green) are shown at the top of each fragMap. The 25- to 40-bp fragments in both IE2-GFP DFF-ChIP data sets mapping over the MIEP core promoter encompassed the TATA box but did not cover the entire *crs*. This protection is consistent with TBP alone binding to the core promoter, which is associated with an approximately 40-bp protection described in our recent study (35). Thus, we refer to this protection as TBP. A subpopulation of slightly larger fragments between 40 and 60 bp in size was observed in both IE2-GFP DFF-ChIP data sets that fully covered the *crs* and was also inclusive of the TATA box (TBP-IE2). IE2 physically interacts with TBP (16), and previous *in vitro* studies have shown that IE2 does not preclude TBP from binding the MIEP *in vitro* (61). Increasing the distance between the TATA box and *crs* interferes with IE2-mediated repression of the MIEP *in vitro* (62). Native DFF-ChIP for TBP revealed a protection at the MIEP that is remarkably similar in size and boundaries to the complex enriched by IE2 (Fig. 4A and B). It was not representative of a TBP-containing preinitiation complex, which protects approximately 70 bp (35). These data support the conclusion that IE2 and TBP cooccupy the repressed MIEP and that IE2 can enrich for a complex that contains TBP engaged at the TATA box while being at least partially dislodged from the *crs*. As additional support for TBP being engaged in a noncanonical complex at the MIEP, we observed that TBP occupancy at the MIEP was extremely high (Fig. S3), similar to that of the highly transcribed RNA4.9, RNA1.2, and RNA2.7 promoters, but the amount of transcription being driven by the IE2-repressed MIEP was comparatively much lower.

In the IE2-GFP and TBP DFF-ChIP fragMaps, a series of complexes larger than TBP or TBP-IE2 were detected (Fig. 4A and B). The largest of these complexes correspond to TBP and IE2 bound with one or two downstream nucleosomes (TBP-IE2-Nuc and TBP-IE2-diNuc), the positions of which are also precisely detected by H3K4me3 DFF-ChIP. The size of the TBP-IE2-Nuc feature suggests that the upstream edge of a nucleosome could encompass the MIEP TSS, the *crs*, and even the TATA box (Fig. 4B, 150-bp line below X-linked IE2 fragMap), which may explain why IE2 appears to be partially dislodged from the *crs*. Interestingly, a major protection extending upstream of TBP-IE2 into the MIEP proximal enhancer region accommodated two previously characterized SP1/SP3 binding sites that are involved in MIEP transactivation (63, 64). The non-cross-linked IE2-GFP DFF-ChIP fragment reveals a number of other unidentified fragment populations enriched by IE2 IP that may reflect additional factors bound within the MIEP proximal enhancer region and the possibly disrupted nature of the promoter-proximal nucleosome. In contrast with patterns detected by probing for IE2, TBP, and H3K4me3, fragMaps portraying total and Ser5P Pol II DFF-ChIP data showed paused Pol II downstream of the MIEP TSS, which protected approximately 40 bp, as well as standard Pol II-containing PICs that protected approximately 70 bp (35). There was little evidence of an association of paused Pol II or the PIC with downstream nucleosomes. These data suggest that viral genomes containing a transcriptionally active MIEP at 48 h are reflected by a very different chromatin structure at this locus than those where the MIEP is repressed by IE2. Taken together, we conclude that IE2 holds the MIEP in a repressed configuration that involves association with TBP and a nucleosome encroaching on the core promoter. Interestingly, IE2 appears to stably occupy the MIEP while only partially engaging the *crs* in this complex, as the *crs* is vulnerable to DFF digestion. A recent structural study reported that yeast TBP can engage the nucleosome at a TATA-like motif close to the end of nucleosomal DNA in the Widom 601 sequence (65), which is possibly similar to the mode of TBP engagement at the MIEP. In this structure, TBP was found to peel away the DNA from the nucleosome. Along this line of thought, TBP disruption of the nucleosome-DNA interface may aid downstream IE2 binding. An envisioned structural model of the MIEP showing the IE2-bound *crs* and TSS in relation the structurally resolved TBP-bound nucleosome is shown in Fig. 4C.

While the profile of IE2 occupancy at the MIEP was noted to be remarkably unique, a subset of other promoters was derepressed upon IE2 depletion, and we investigated whether a related mechanism of repression was at play. As an example, the UL144 promoter exhibits a significant increase in paused Pol II PRO-Seq signals upon IE2 depletion, and indeed, we found that IE2 engaged a consensus motif within its core promoter region (Fig. 4D). FragMap analysis of IE2 occupancy at the UL144 promoter, in addition to the US29 promoter, which is also repressed by IE2, indicated a potential enrichment for up- or downstream-associated nucleosomes (Fig. S4A). Extending our analyses to all remaining promoters derepressed at least 2-fold upon IE2 depletion, we observed that IE2 invariably engages a consensus sequence within the core promoter region proximal to the TSS, as graphically depicted on the left in Fig. 4E. Interestingly, a subset of these IE2-repressed promoters is expressed with late kinetics, as indicated by the high sensitivity of their promoter activity to treatment of infected cells with phosphonoformic acid (PFA) (Fig. 4E, right). PFA is an HCMV replication inhibitor that prevents the transcription of late genes (12). The PFA sensitivity index (PSI), defined by Li et al. (12), measures the ratio of paused Pol II PRO-Seq reads at 72 hpi in control cells and in cells treated with PFA from the onset of infection. The US29 and putative ORF249LC (4) promoters, for example, have very high PSI values and exhibit markedly increased activity at 48 hpi (postreplication onset) in an HCMV time course PRO-Seq experiment compared to the UL112/113 promoter, which became active very early in infection (Fig. S4B). Transcription of late genes is orchestrated by a set of LTFs, one of which, UL87, substitutes for TBP in a specialized viral Pol II preinitiation complex and preferentially recognizes distinct upstream core promoter elements, including TATT and TATAT (13). Both the US29 and ORF249LC promoters contain an upstream TATT, and native DFF-ChIP for UL87 at 48 hpi revealed its association with these promoters (Fig. 4F). Thus, it appears that IE2-mediated repression, previously linked only to the MIEP, extends as well to other TBP- and UL87-driven promoters through a similar mechanism involving direct engagement of the core promoter.

10.1128/mbio.00337-22.5 FIG S4 Extended analysis of IE2-mediated repression. (A) FragMaps for the UL144 (top) and US29 (bottom) promoter regions. Fragments corresponding to bound IE2 and potentially associated adjacent nucleosomes are indicated, as are the major TSSs. (B) PRO-Seq datasets for an HCMV time course showing a strong induction of the US29 and ORF249LC promoters at 48 hpi, postreplication onset, compared to the UL112 promoter, which is activated early in infection. Download FIG S4, PDF file, 0.3 MB. Copyright © 2022 Ball et al. 2022 Ball et al. https://creativecommons.org/licenses/by/4.0/ This content is distributed under the terms of the Creative Commons Attribution 4.0 International license.

### IE2-mediated transactivation is associated with binding nearby target core promoter regions.

**FIG 5:**
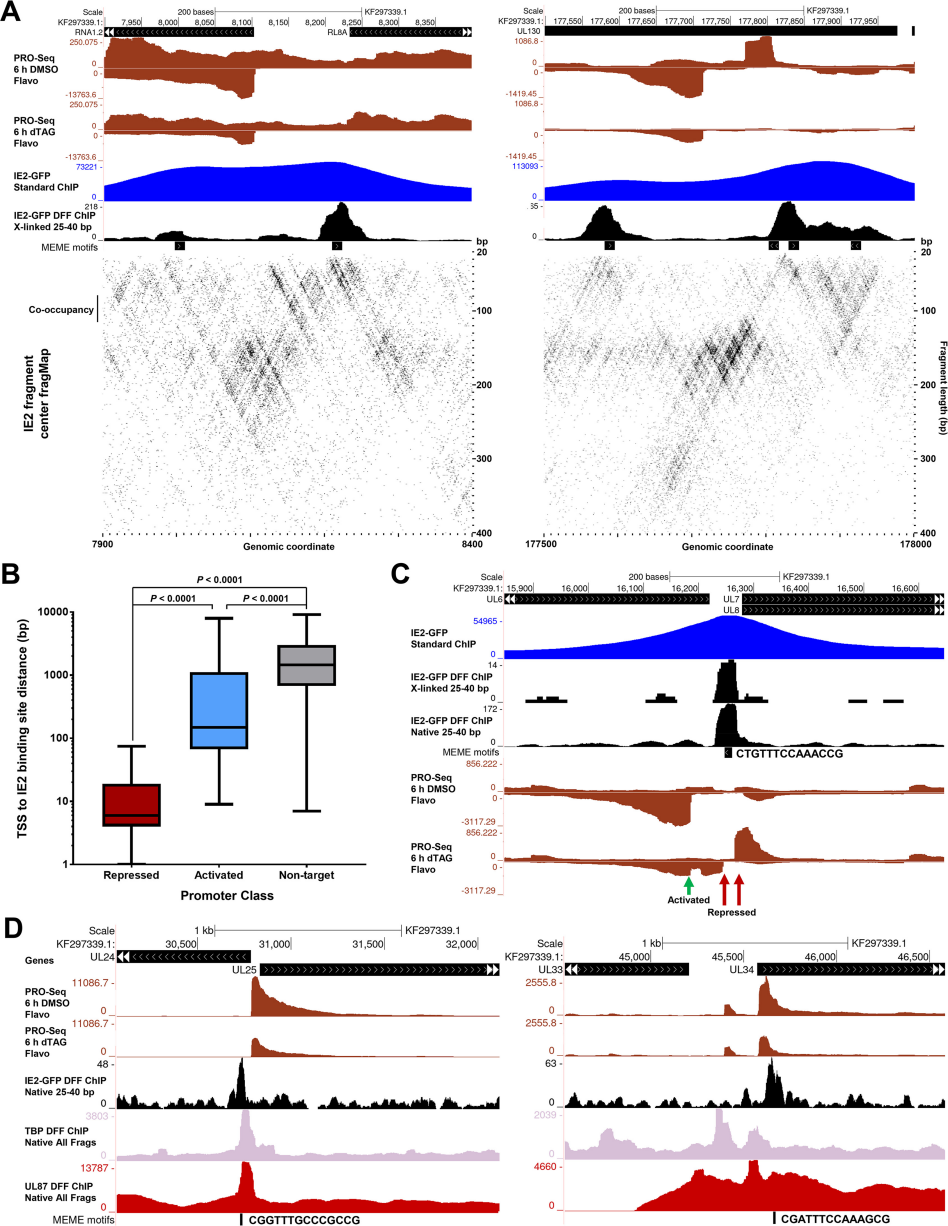
IE2 activates transcription through engagement of sites nearby but not within core promoter regions. (A) Genome browser views of PRO-Seq data (TB40/E IE2-FKBP 72 hpi Flavo, with or without dTAG) at the RNA 1.2 promoter region and over several active promoters within the UL130 locus that are activated by IE2. Standard IE2-GFP ChIP-Seq and IE2-GFP DFF-ChIP reveal promoter-proximal sites of IE2 occupancy correlating with MEME consensus motifs. Below these regions, fragment-center fragMaps plotting fragments 20 to 400 bp in size reveal the positions of sites directly bound by IE2 and show evidence of larger fragments spanning two adjacent binding sites. (B) Boxplot analysis of distances between the maximum TSS of promoters repressed (*n* = 7) or activated (*n* = 45) by IE2 and maximum TSS of promoters unaffected by IE2 (*n* = 473) and the nearest IE2 binding motif. Repressed promoters were defined as having a ratio of PRO-Seq 5′ ends (TB40/E IE2-FKBP, 72 hpi, dTAG/DMSO) greater than 2 (defined in Li et al. [12]), activated less than 0.5, and nontarget between 0.8 and 1.2. Significance was determined using a Mann-Whitney test. (C) Demonstration of the distance-dependent effects of IE2 on transcription. IE2 binding within the UL7/8 promoter region at one site simultaneously represses the indicated promoters on the forward and reverse strands and activates a more distal promoter on the reverse strand. (D) Examples of IE2 binding immediately up- and downstream of TBP and/or UL87 PICs formed at the IE2-activated UL25 and UL34 promoters.

IE2 primarily functions as an activator in late infection, stimulating transcription from many early-late and late promoters that were previously discovered to be largely absent from upstream TATT elements (12). Investigation of IE2-GFP DFF-ChIP signals at activated promoter regions suggested that IE2 binds to consensus motifs that are close by but not overlapping the TSS. For example, the RNA1.2 promoter exhibits IE2 binding both up- and downstream of the TSS (Fig. 5A, left). The upstream IE2 occupancy correlated with previously published IE2 DNase protection analyses for this promoter region (26). Several promoters within the UL130 locus exhibited a collapse in transcriptional activity following IE2 depletion, and the IE2-GFP DFF-ChIP data here suggest that numerous close-by sites in the region are engaged by IE2 (Fig. 5A, right). It is possible that IE2 engages multiple nearby sites simultaneously and operates synergistically to stimulate transcription. To examine this, fragment center fragMaps were generated for the RNA1.2 and UL130 regions. These plots are similar to fragMaps, but the density of fragment centers is plotted instead of the entire fragment. An enrichment of longer fragments with ends that encompass two or more adjacent binding sites would be indicative of cooccupancy. However, the ability to detect such events would correlate negatively with the distance intervening two sites due to the increased likelihood of DFF digestion between them. A slight enrichment of fragments up to approximately 100 bp that encompassed two adjacent and potentially cooccupied IE2 binding sites was detected, indicating that DFF-ChIP may be able to inform on transcription factor cooccupancy (Fig. 5A, bottom fragMaps).

To better understand the constraints on target promoter regulation as it relates to IE2 binding, the distance between the maximum TSS of every promoter repressed or activated at least 2-fold by IE2 and the nearest IE2 binding site was calculated. These distances were compared in a boxplot to the distances between the maximum TSS of promoters mostly unaffected by IE2 depletion (nontarget, 0.8- to 1.2-fold change) and the nearest IE2 binding site (Fig. 5B and Table S1; see also Materials and Methods). As previously shown, IE2 bound nearly on top of the TSS of repressed promoters. IE2-activated promoters tend to be significantly closer to bound IE2 than nontarget promoters, suggesting that distance to the target promoter is a constraint for IE2 transactivation. This paradigm was well-represented by the UL7/8 promoter region, wherein IE2 bound at one site simultaneously represses two oppositely oriented promoters overlapping the IE2 binding site and activates a more distal promoter on the reverse strand (Fig. 5C). IE2 apparently can activate transcription by binding either up or downstream of the PIC, as evidenced by IE2 DFF-ChIP signals at the UL25 and UL34 promoter regions (Fig. 5D). IE2 binds a motif immediately upstream of the PIC at the UL25 promoter and immediately downstream of the PIC at the UL34 promoter. UL25 encodes a tegument protein that is expressed with late kinetics, and recent UL87 depletion studies reveal that it is substantially dependent on UL87 protein. The core promoter contains an upstream TATAT element that was associated with UL87 recruitment. DFF-ChIP for TBP and UL87 reveal that the UL25 promoter utilizes both PIC types, which raises the possibility that IE2 stimulates the formation of either. UL34 transcription is directed by at least two promoters. In late infection, IE2 specifically stimulated transcription from the closer downstream promoter. Regarding a mechanism for activation, it is possible the IE2 binding proximal to the core promoter region recruits initiation machinery, as IE2 is known to interact with TBP, TFIIB, and other subunits of TFIID (16, 21).

### IE2 directly functions as a roadblock to Pol II elongation.

**FIG 6:**
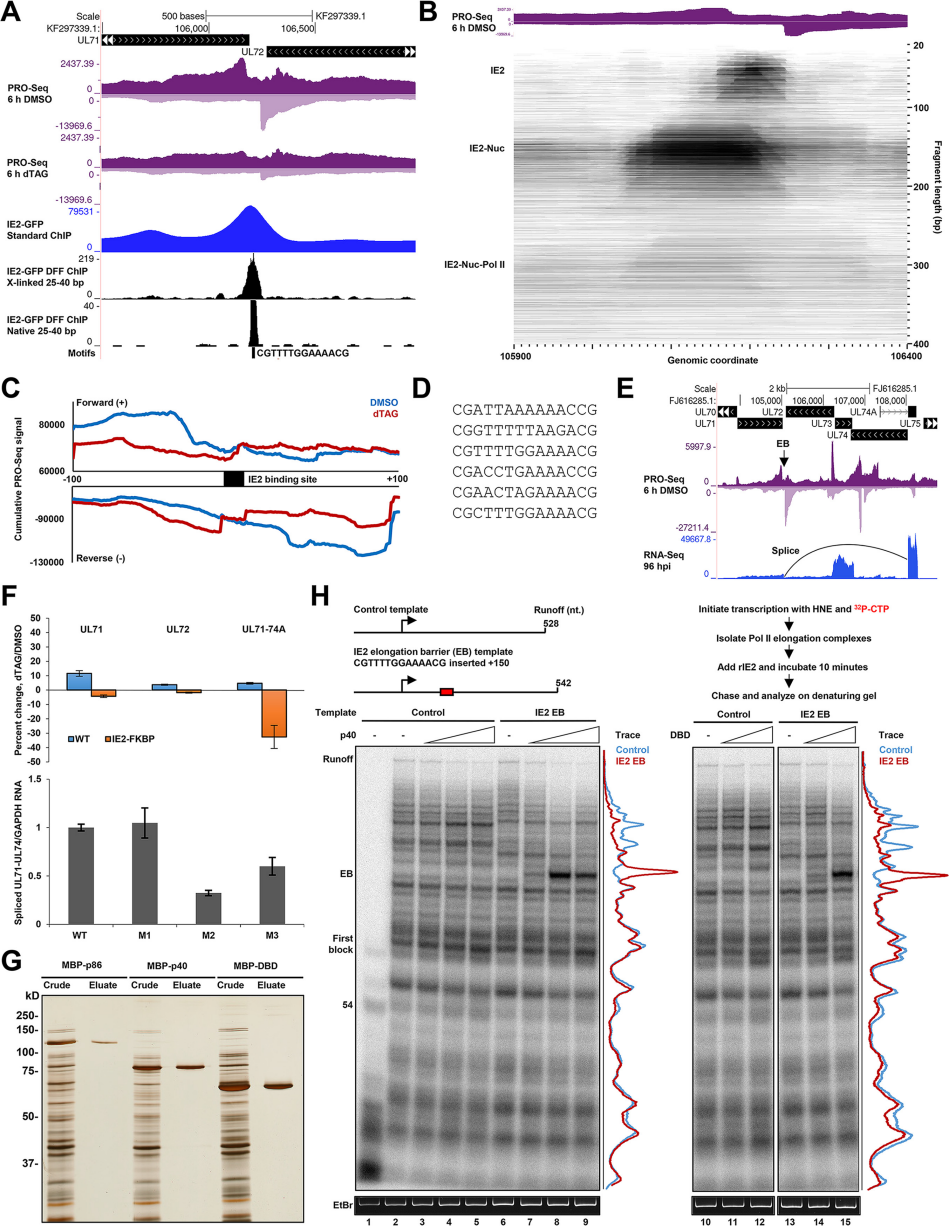
IE2 functions as a direct barrier to Pol II elongation. (A) Genome browser view of PRO-Seq data (72 hpi TB40/E IE2-FKBP DMSO, with or without dTAG) showing an IE2-mediated barrier to Pol II elongation and IE2 binding to a consensus site located between the bidirectional pileups of PRO-Seq signal. (B) FragMap of cross-linked IE2-GFP DFF-ChIP data at the elongation barrier-associated region shown in panel A, revealing an adjacently associated nucleosome on engaged IE2. (C) Metaplots of sense and antisense PRO-Seq signal ±100 bp relative to identified IE2 consensus sites with and without 6-h dTAG treatment. (D) Examples of motifs associated with IE2 elongation barrier function. (E) Genome browser view of Towne IE2-FKBP PRO-Seq data at 96 hpi and RNA-Seq data at 96 hpi, indicating the splice from the UL71 donor site to the downstream acceptor site at the UL74A second exon in relation to the IE2 elongation barrier. (F, top) RT-qPCR data showing levels of relative levels of UL71, UL72, and UL74-74A mRNA quantified in proportion to GAPDH in cells infected with wild-type virus or IE2-FKBP virus and treated for 6 h with DMSO or dTAG. (Bottom) RT-qPCR data reporting on levels of spliced UL71-74A/GAPDH RNA in cells infected with wild-type virus or viruses harboring the indicated mutations in the IE2 elongation barrier-associated consensus site. (G) Silver stain of crude lysates and nickel eluates for the indicated recombinant MBP- and 6×-His-tagged IE2 proteins. (H, upper) Schematics of *in vitro* transcription templates and flow chart describing assay. (Lower) Phosphor images of labeled nascent transcripts generated during *in vitro* transcription. A lane profile analysis of signals for the middle IE2 p40 titration point and greater DBD titration point are shown to the right of the gels. Transcripts associated with runoff, the specific elongation barrier, and the early block are indicated. Ethidium bromide staining of template DNA shows relative levels of template recovery for each reaction.

Our analyses of IE2 depletion PRO-Seq data uncovered a third and novel function of IE2 as an elongation barrier. Examination of IE2 ChIP signals at the representative locus between the UL71 and UL72 genes revealed that IE2 engaged a nearly palindromic consensus site centered approximately 25 bp downstream of the bidirectional pileups of PRO-Seq signal representing productively elongating Pol II (Fig. 2F and 6A). These data suggest that IE2 functions as a direct obstacle to Pol II elongation in cells. FragMap analysis of this locus further revealed that IE2 enriched for a nucleosome adjacently positioned to one side, and evidence exists for an H3K4me3 nucleosome as this position (Fig. 6B and Fig. S3). Transcription along the reverse strand encounters IE2 before the nucleosome and seemed to be more sharply impacted by the IE2 barrier. Investigation of other barrier sites revealed the multiple contexts in which this effect of IE2 was observed. For example, IE2 transcriptional barriers were found within an intron in the UL89 gene, at the UL144 promoter region where IE2 also functions as a repressor, and at the end of the UL112 gene. Of note, the IE2 binding site at the end of the UL112 gene, CGATTAAAAAAC**A**G, contains a single C-to-A substitution (boldfaced position) in TB40/E that would likely reduce IE2 affinity for this site based on previous *in vitro* studies (29). Correspondingly, the Towne virus, which contains CG dinucleotides at both ends of the binding site, was associated with a much stronger transcriptional barrier effect (Fig. S5A). Regions associated with IE2 elongation barrier function were conserved across HCMV strains, including clinical-like strains such as Merlin (Fig. S5B). To demonstrate the commonality of IE2 barrier function, meta-analysis of forward- and reverse-strand PRO-Seq data ±100 bp relative to 78 defined IE2 binding sites revealed a bidirectional pile of signal upstream of the binding site that diminished with IE2 depletion (Fig. 6C). Downstream signals were modestly increased following IE2 depletion, suggesting that polymerases that would have been stalled by IE2 instead elongated past the site of the barrier when IE2 is removed. Elongation barrier function tended to be associated with highly symmetrical and/or AT-rich binding sites (examples taken from various contexts in Fig. 6D), which are likely to be high-affinity sites for IE2 based on previous *in vitro* studies (29, 47).

10.1128/mbio.00337-22.6 FIG S5 Extended analysis of IE2 elongation barrier function. (A) Examples of IE2 elongation barriers. The center of the barrier is indicated by an arrow, and IE2 occupancy and the corresponding consensus motifs are indicated. (B) Conservation analysis of a 114-bp region centered on the UL71 and UL72 elongation barrier-associated IE2 binding site across various laboratory and clinical-like HCMV strains. (C) Designation of the IE2 binding site mutants generated to study the function of the IE2 elongation barrier at the UL71 and UL72 locus. (D) qRT-PCR data at 72 hpi showing unchanged levels of UL71 and UL72 mRNA relative to GAPDH in wild-type and binding site mutant viruses. (E) MBP-IE2 DBD silver stain EMSA showing a laddered pattern of IE2-DBD in the absence of DNA and a shift with the addition of a dsDNA probe representing a perfectly palindromic, ideal IE2 binding site. Asterisks indicate the differentially migrating forms of IE2 observed in the absence and presence of DNA. (F) *In vitro* transcription assays showing that IE2 p86 blocks Pol II elongation on a template in a reaction context identical to those shown in Fig. 6H and that IE2 p40 partially inhibits Pol II elongation on the template in the presence of all factors in crude nuclear extract. Download FIG S5, PDF file, 1.9 MB. Copyright © 2022 Ball et al. 2022 Ball et al. https://creativecommons.org/licenses/by/4.0/ This content is distributed under the terms of the Creative Commons Attribution 4.0 International license.

Initial analyses of RNA-Seq data collected after brief depletion of IE2 revealed no major effects on expression of the UL71 and UL72 mRNAs, which are associated with a prominent IE2 elongation barrier (Fig. 1F and 6A). In follow-up, it was noted that the UL71 transcript splices to the second exon of the UL74A gene, as shown in Fig. 6E. Pol II elongation kinetics have been tightly linked to splicing outcomes, with one model being that Pol II pausing can enable the recognition of weak splice sites during cotranscriptional processing (66, 67). Therefore, it was tested whether IE2 depletion impacted splicing of the UL71-UL74A transcript through reverse transcription-quantitative PCR (RT-qPCR) assays that individually measured the abundances of the UL71, UL72, and UL71-74A RNAs relative to a host glyceraldehyde-3-phosphate dehydrogenase (GAPDH) reference RNA. Depletion of IE2 for 6 h negligibly impacted the levels of UL71 and UL72 mRNA but reduced the level of UL71-UL74A spliced RNA (Fig. 6F). To further test, Towne mutants in which the barrier-associated IE2 binding site was mutagenized were generated (Fig. S5C). Compared to wild type, the mutant viruses exhibited no significant change in UL71 and UL72 mRNA levels at 72 hpi (Fig. S5D). Mutation of the upstream CG dinucleotide did not substantially impact the levels of the UL71-UL74A RNA. However, mutations that additionally reduced the T-richness of the upstream half-site or converted the IE2 binding to the *crs* resulted in decreased levels of the UL71-UL74A RNA compared to the wild-type virus. These data suggest that IE2 elongation barrier function promotes the splicing of the UL71 RNA to the second exon of UL74A. A caveat to these experiments is that the UL71-74A donor splice site is encoded within the IE2 binding site, at the downstream **C**G dinucleotide (boldfaced C splices to UL74A acceptor), complicating the mutational approach. In addition, reduced levels of UL71-74A RNA were not observed at assayed time points in RNA-Seq data sets, which may relate to incomplete IE2 depletion. We note that a previous study investigating the effects of miRNA-mediated suppression of IE2 in fibroblasts infected with HCMV at a low multiplicity of infection (MOI) for 9 days showed significant reductions in levels of both UL71 and UL74A mRNA (68), suggesting that the ability to detect changes in expression of these possible IE2 target genes vary with respect to MOI or the timing and duration of IE2 depletion.

To test whether IE2 alone is sufficient to function as an elongation barrier, recombinant versions of IE2 p86, IE2 p40, and the core DBD (amino acids [aa] 346 to 579) fused to MBP and a 6× His tag were expressed in Escherichia coli and purified for use in *in vitro* transcription assays (Fig. 6G and H). Silver staining of nickel eluates indicated that the protein preparations were highly pure (Fig. 6G). The purified DBD was analyzed by silver-stain electrophoretic mobility shift assay (EMSA) for binding to a perfectly palindromic, ideal IE2 binding site, CGGTTTGCAAACCG, derived from the consensus MEME motif. Interestingly, the DBD in the absence of DNA migrated as a ladder, which may relate to previously reported oligomerization of IE2 (28, 29). As expected, the DBD shifted with addition of the palindromic DNA probe (Fig. S5E). A biotinylated *in vitro* transcription template driven by the CMV promoter was modified such that the native *crs* was extensively mutated to abrogate IE2 binding to the core promoter region. This control template was further modified to introduce the UL71/UL72 elongation barrier-associated IE2 binding site CGTTTTGGAAAACG 150 bp downstream of the TSS (IE2 EB template). To test whether IE2 can block Pol II elongation on the template in a manner that requires the IE2 binding site, transcription was initiated on the control and IE2 EB templates immobilized to streptavidin beads with crude HeLa nuclear extract in the presence of [α-^32^P]CTP. Early Pol II elongation complexes containing radiolabeled nascent RNAs were isolated by washing the immobilized complexes in high salt. Reaction mixtures were incubated with increasing amounts of IE2 p40 or the DBD and subsequently chased for 20 min (Fig. 6H). A no-chase lane shows the profile of transcripts associated with early elongation complexes (Fig. 6H, lane 1). Addition of p40 and the DBD into the IE2 EB template reactions, but not the control template reactions, led to a block in Pol II elongation down the template during the chase (compare lanes 2 to 5 with 6 to 9 and 11 to 12 with 13 to 15; also see adjacent lane profiles). The effect could be titrated, and it was noted that the largest amounts of p40 and DBD induced an early, weak block to elongation upstream on both templates. This effect is potentially associated with a CG dinucleotide followed by an A-rich stretch in the template. Parallel experiments carried out with purified IE2 p86 showed similar results (Fig. S5F, left). Pol II in elongation complexes isolated under these conditions exhibits a slow elongation rate, as productive elongation factors in nuclear extract are not present during the chase. Thus, it was tested whether IE2 p40 could inhibit Pol II elongation in the presence of all factors in crude extract by titrating IE2 p40 into reaction mixtures prior to initiation. Pulse-chase experiments revealed a modest accumulation of Pol II at the barrier site in this reaction context as well (Fig. S5F, right). Taken together, these data establish that IE2 can directly function as a partial barrier to elongation *in vivo* and *in vitro* and that this function may be important in one context for accurate splicing of a viral RNA.

### A unique profile of IE2 engagement at HCMV *ori*Lyt.

HCMV *ori*Lyt is the site of initiation of viral DNA synthesis. Previous studies have identified two essential regions within *ori*Lyt and core HCMV replication machinery (69–71). In addition to this core machinery, IE2 and the UL84 protein are required for viral genome replication (72–74). However, their roles in this process remain unclear. IE2 has been suggested to function as an origin-binding protein that may promote assembly of the replication machinery, possibly by way of activating a promoter within *ori*Lyt (73).

**FIG 7:**
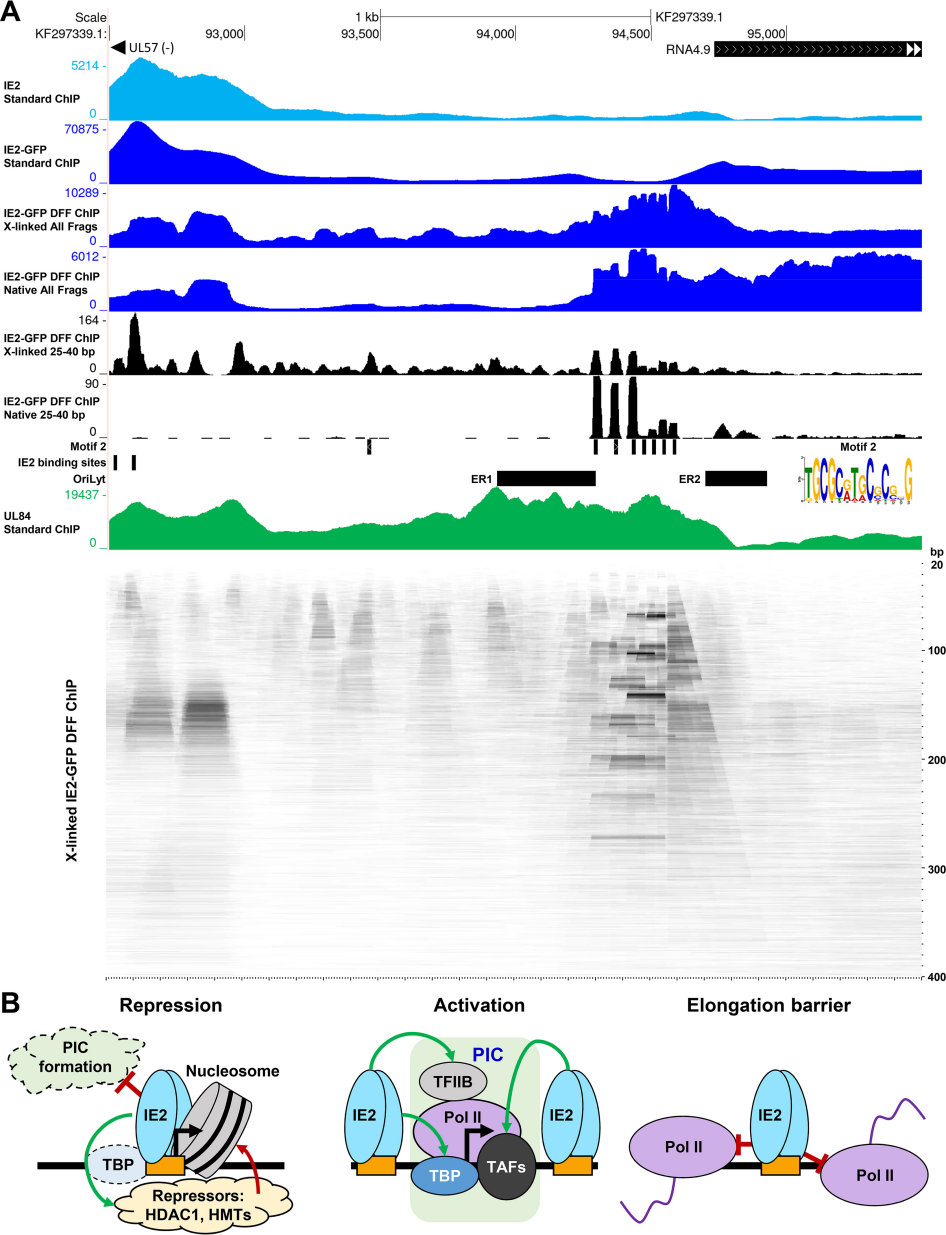
Unique profile of IE2 occupancy at *ori*Lyt. (A) Genome browser view of previously published IE2 ChIP (TB40/E 72 hpi), standard IE2-GFP ChIP-Seq, cross-linked and native IE2-GFP DFF-ChIP (all fragments and 25 to 40 bp, 48 hpi), and previously published UL84 ChIP (TB40/E 72 hpi) in the *ori*Lyt region. Essential regions I and II within *ori*Lyt are indicated. The locations of a novel motif 2 and consensus IE2 binding sites in *ori*Lyt are indicated. Below is an aligned fragMap of the *ori*Lyt region representing the same region shown in the genome browser track. (B) Model representing the salient findings regarding mechanisms of IE2 function in late infection. IE2 functions as a repressor, activator, and elongation barrier during late infection, and each function is linked to IE2 binding predominantly at consensus motifs. IE2 generally represses transcription by binding to core promoters and interfering with PIC assembly. At the MIEP, IE2 is bound in the context of a nucleosome that encroaches on the core promoter. Here, the repressed state of the MIEP is likely reinforced by IE2-mediated recruitment of HDAC1 and HMTs. IE2 activates transcription through engagement of sites proximal to target core promoters, possibly facilitating formations of PICs through interactions with TBP, TFIIB, and TAFs. Finally, IE2 can function as a direct, bidirectional obstacle to Pol II elongation.

Examination of IE2 ChIP data in the *ori*Lyt region revealed a unique pattern of occupancy (Fig. 7A). Recently reported IE2 ChIP-Seq for TB40/E performed at 72 hpi (40) documented a broad peak of occupancy in the UL57 promoter region upstream of *ori*Lyt and a trough of signal over essential region I and in the space intervening essential regions I and II. Our standard ChIP for IE2-GFP at 48 hpi reproduced this pattern. In contrast with these results, both cross-linked and native IE2-GFP DFF-ChIP showed an array of sharp pillar-like signals in a region overlapping partially with essential region I and extending toward the RNA4.9 promoter. In the cross-linked IE2-GFP DFF ChIP data sets, IE2 engagement was observed across the entire *ori*Lyt (Fig. 7A and Fig. S3). These peaks were not associated with an IE2 consensus motif, and none were predicted by FIMO. However, the second-scoring motif in our MEME analysis of regions called by IE2 DFF-CSP mapped squarely back to these peaks (Fig. 7A, motif 2).

Our data indicated a clear discrepancy in the results provided by standard ChIP and DFF-ChIP. In addition, the absence of the IE2 consensus sites at *ori*Lyt suggested that occupancy detected by DFF-ChIP arose from an alternative binding mode. Standard ChIP was carried out under mildly denaturing conditions with sonication. Proteins that are not directly associated with DNA are often difficult to detect by ChIP due to an absence of protein-DNA cross-links and may be lost from chromatin-bound complexes under harsh conditions. In contrast, DFF-ChIP was carried out under gentle conditions amenable to DFF digestion that may retain signals associated with factors not directly bound to DNA. These methodological differences may relate to the discrepant patterns of IE2 occupancy that were observed.

The UL84 protein has also been suggested to function as an origin binding protein (75), and IE2 and UL84 interact throughout lytic infection (76). Recently reported UL84 ChIP-Seq from cells infected with TB40/E for 72 h shows a broad enrichment of UL84 occupancy over *ori*Lyt, as noted by the authors (40) (Fig. 7A). This enrichment of UL84 correlates with sites engaged by IE2 in the IE2-GFP DFF-ChIP data. Thus, it is possible that the pattern of signals observed in IE2-GFP DFF-ChIP data stems from UL84 engagement of *ori*Lyt and indirect association by IE2. Furthermore, our analysis identified a motif that may be associated with UL84 binding (motif 2). FragMap analysis of cross-linked IE2-GFP DFF-ChIP data in this region revealed an array of sites between essential regions I and II that engaged by IE2 and evidently cooccupied, as substantially larger fragments representing multiple peaks within the region were observed (Fig. 7A, lower). These data suggest that a large and stable protein-DNA complex involving IE2 and UL84 exists at *ori*Lyt.

## DISCUSSION

Here, we report that IE2 operates as a multifunctional Pol II repressor, activator, and elongation barrier during late HCMV infection. Mechanistically, IE2-mediated control of transcription was linked to its direct engagement of numerous consensus sites on the viral genome. The effect of IE2 on transcription was highly context dependent, varying in relation to the distance between IE2 binding sites and target promoter regions and interactions between bound IE2 and Pol II elongation complexes. Implementation of DFF-ChIP, a method recently developed by our lab, demonstrated its utility for defining transcription factor occupancy at high resolution and informing on their local chromatin environment. DFF-ChIP revealed potentially functional interactions between IE2 and neighboring nucleosomes, which is highlighted at the MIEP. A model that summarizes our salient findings regarding mechanisms of IE2 function in transcriptional control and integrates existing knowledge is shown in Fig. 7B.

Our study unambiguously demonstrated that IE2 engages numerous consensus sites on viral chromatin during late infection. Genome-wide investigations of IE2 occupancy on the host and viral genomes were not previously performed prior to our study. A consensus motif for IE2 was determined through three parallel peak-finding and MEME analyses of IE2-GFP ChIP-Seq data sets, one of which utilized a novel peak-calling algorithm, DFF-CSP, that we developed and optimized for the analysis of DFF-ChIP data. IE2 was found not to functionally engage the host genome, and sequencing reads in IE2 ChIP data sets disproportionately mapped to the viral genome. Two properties of viral chromatin may explain the preferential association of IE2 with the HCMV genome during late infection. First, the HCMV genome is broadly accessible. This is in stark contrast to the host genome, where nucleosome occupancy is generally high outside accessible regulatory regions. Second, the HCMV genome exhibited a much higher density of IE2 consensus matches than the host genome. Approximately 1,200 sites similar to the IE2 consensus were found on the HCMV genome with FIMO, whereas approximately 90,000 matches were detected on the host genome. This equates to approximately five IE2 binding sites per kilobase pair on the HCMV genome and 0.02 sites per kilobase pair on the host genome. A seeming conundrum is that despite the presence of a large number of potential IE2 binding sites on the HCMV genome, only a fraction of them were occupied (495 DFF-CSP peaks and 78 MEME consensus motifs). This indicates that other variables, such as high levels of transcription or blockage of sites by other proteins or complexes, influence IE2 occupancy. Previous immunofluorescence studies of IE2 in HCMV-infected cells suggested that IE2 predominantly localizes to the viral replication compartment in late infection and is enriched near viral genomes at PML bodies even prior to replication onset (37, 77, 78). This localization was also dependent on the ability of IE2 to bind DNA (37). The reported propensity for IE2 to form multimers *in vitro* and *in vivo* might also impact IE2 subnuclear localization and diffusion properties (28, 29).

Existing models of IE2 function suggest that it operates as a promiscuous transactivator of viral early genes. We did not investigate IE2 binding to the viral genome during early infection, but it was noted that recently published IE2 ChIP-Seq performed at 20 hpi (40) did not correlate with ChIP-Seq data sets generated for IE2 at 48 or 72 hpi. The analogous herpes simplex virus 1 immediate-early transactivator ICP4 was recently shown to exhibit broad association with the HSV genome during early infection, possibly facilitated by the formation of ICP4 oligomers, while ICP4 occupancy during late infection resolved to sharper peaks over ICP4 consensus binding sites through a proposed mass action mechanism implicating the ratio of ICP4 protein to viral DNA (46). A similar model may be invoked for HCMV, wherein IE2 p86 broadly samples viral chromatin during early infection, while during late infection increasing amounts of viral genomic DNA emphasize IE2-mediated transcriptional control through engagement of consensus binding sites.

IE2 functions as a repressor by binding within core promoters to inhibit PIC assembly. At the MIEP, IE2-GFP DFF-ChIP revealed an unexpected architecture involving IE2 binding to the core promoter region in association with downstream nucleosomes and upstream TBP. Strikingly, close inspection of the data revealed that the most promoter-proximal nucleosome encroached on and buried the TSS. At most active host Pol II promoters, a well-positioned +1 nucleosome has an upstream edge located approximately 50 bp downstream of the maximum TSS (53, 79). Important contacts between the Pol II preinitiation complex and DNA occur up to 30 bp downstream of the TSS (53). Thus, the IE2-repressed MIEP is packaged in a manner highly refractory to transcription initiation. A recent study reported that at very early times postinfection, the MIEP is enriched for activating H3K27ac, whereas at later times (24 hpi) associated with IE2 p86-driven autorepression, the MIEP exhibited a gain in repressive H3K27me3 (56). Several other studies have reported on epigenetic control of the MIEP during lytic infection, demonstrating the presence of activating and repressive histone modifications (59, 80), the recruitment of repressive chromatin-modifying complexes to the MIEP by IE2 (19), and the induction of the MIEP by histone deacetylase (HDAC) inhibitors (19, 81). H3K4me3 nucleosomes detected in association with IE2 at the MIEP in this study may also bear repressive modifications, corresponding to a bivalent state. Finally, our investigations of IE2-repressed promoters revealed that IE2 not only represses transcription driven by canonical host TBP-containing PICs but also represses transcription driven by UL87 viral PICs on late promoters.

IE2 activates transcription from a subset of HCMV promoters during late infection by binding nearby core promoter regions but not within them. Very interestingly, both effects can be elicited by IE2 binding at a single site (Fig. 5C). Although a defined mechanism of activation is not yet clear, we speculate that known interactions between IE2 and GTFs are important and facilitate the recruitment of the transcriptional machinery. Here, transgenic complementation of wild-type IE2 proteins and mutants that disrupt IE2-GTF interactions in the context of viral IE2-FKBP depletion may provide clarity. In addition, possible differential involvement of IE2 isoforms in transcriptional regulation can be addressed in this way. Further analysis of IE2 DFF-ChIP data revealed that some loci contain multiple close-by sites of IE2 occupancy that may operate synergistically to drive local transcription initiation. These crowded profiles of IE2 occupancy were impossible to visualize in standard ChIP-Seq data due to its comparatively poor resolution.

Our data favor a model in which IE2-mediated activation during late infection is associated with sequence-specific IE2 DNA binding to promoter-proximal sites. However, it was noted that not all IE2-activated promoters exhibit strong evidence of IE2 bound to a consensus site in their vicinity. For example, the highly IE2-dependent UL83 promoter exhibits a weakly occupied IE2 binding site downstream of the TSS. It is possible that IE2-mediated transactivation occurs over greater genomic distances on the HCMV genome, as is thought to be common on the host genome (82). Other modes of IE2-mediated transactivation have been reported. For example, a previous study has suggested that IE2 indirectly activates transcription through interactions with GTFs, behaving as a TAF-like factor (21). In this model, one can expect to see IE2 DFF-ChIP profiles that generally correlate with those of TBP, but this was not apparent (see Fig. S3 in the supplemental material). Furthermore, specificity is not conferred by this model, and IE2 clearly regulates transcription from a minority of promoters during late infection. Other studies have suggested that IE2 functions through protein-protein interactions with host transcription factors, in conjunction with sequence-specific IE2 DNA binding (24–26). Our results are congruent with the idea that IE2 and host TFs cooperate and do not exclude a role for protein-protein interactions but underscore the importance of IE2 DNA binding nearby activated promoters during late infection.

IE2 functions as a direct barrier to Pol II elongation *in vivo* and *in vitro*. This previously unreported function was linked to IE2 binding at consensus sites that exhibited a relatively high degree of symmetry and/or AT richness. An elongation block between the UL71 and UL72 genes was found to be important for splicing of the UL71-74A RNA. This may be analogous to a previously reported function of host CTCF, a tight-binding transcription factor that has been shown to promote the inclusion of upstream exons by impeding Pol II elongation (67). Apart from the promoter-proximal pausing factors DSIF and NELF, in addition to intrinsic causes of pausing, such as nucleosomes and certain DNA sequences, CTCF and IE2 are the only transcription factors of which we are aware that block Pol II elongation in a native biological context. This function of IE2 was reconstituted *in vitro* in the absence and presence of Pol II productive elongation factors. Notably, the elongation rate of Pol II *in vivo* is inconstant and regulated (66, 83). An unaddressed question is whether elongation rate control is important for Pol II susceptibility to IE2 barrier function in cells. Elongation barriers were observed in multiple genomic contexts: between convergent genes, within gene bodies, and at promoter regions. It is possible that elongation barriers tune gene expression by slowing Pol II elongation through genes. Alternatively, barriers may dampen conflicts between convergent transcription units or insulate promoter regions from encroaching elongation complexes, two functions that may benefit accurate gene expression on a crowded and highly transcribed HCMV genome.

This work greatly extends our understanding of transcriptional control by IE2 during late HCMV infection and identifies numerous functional IE2 binding sites on the viral genome that can be further dissected in future studies. Our use of DFF-ChIP highlights its utility in investigating transcription factor and nucleosome interactions and their dynamics. The use of DFF-ChIP may be helpful in understanding how IE2 samples the viral genome during reactivation from latency, a state in which the viral genome is thought to be packaged as heterochromatin (80). The improved understanding of IE2-mediated transcriptional control provided by this study will greatly benefit the ongoing development and validation of antiviral drugs targeting HCMV immediate-early proteins (84, 85).

## MATERIALS AND METHODS

### Cells and viruses.

Deidentified discarded human foreskins were used for the isolation of HFF, in accordance with University of Iowa IRB number 201702734. HFF were maintained in minimum essential medium supplemented with heat-inactivated 5% fetal bovine serum and 1,000 U/mL penicillin-streptomycin. All experiments were carried out with cells at passage number ≤6. Construction of the HCMV TB40E IE2-FKBP and HCMV Towne IE2-FKBP viruses was previously documented in Li et al. (12). A previously developed TB40/E mCherry bacterial artificial chromosome (BAC) (86) (gift of Eain Murphy to Jeffery Meier) was used to derive the TB40/E IE2-GFP virus. Nucleic acid reagents that were utilized for construction of the TB40/E IE2-GFP virus are listed in the Data Set S1 in the supplemental material. Briefly, the bacterial *galK* gene was placed at the C terminus of the UL122 open reading frame (ORF). The *galK* gene was then replaced by DNA encoding enhanced GFP (eGFP) such that it was fused in-frame at the C terminus of UL122. The BAC was validated by Sanger sequencing and restriction fragment length polymorphism analysis. Modified BAC DNA was nucleofected into HFF using an Amaxa neonatal human dermal fibroblast kit (VPD-001; Lonza) and an Amaxa Nucleofector II with program setting U23. Infected HFF were mixed with ARPE-10 cells, and infected ARPE-19 cells were aliquoted and stored for later inoculation of HFF to make viral stocks for experiments. Viral replication cycles in HFF were kept at a minimum to maintain the phenotypic and genotypic characteristics of HCMV TB40/E IE2-GFP. Viral stocks used for experiments were prepared from the supernatants of infected HFF. The supernatant was passed through a 0.45-μm filter, and the virus was pelleted through a 20% sorbitol cushion. Viral titers were determined by serial dilution into a 24-well plate of HFF and examined by immunofluorescent assay of HCMV IE1 and IE2 protein at 24 hpi using monoclonal antibody MAB810 (1:1,000; EMD Millipore) and secondary goat anti-mouse IgG (H+L) antibody conjugated to Alexa Fluor 555 (A-21422; ThermoFisher Scientific). DAPI counterstain of nuclei was utilized to determine the percentage of infected cells.

### Western blotting.

Whole-cell lysates were prepared in 1× SDS protein sample buffer on ice and subjected to 4 rounds of sonication (10 s on, 10 s off, 40% amplitude) in a QSonica sonicator water bath chilled to 4°C. Proteins were separated by 10% SDS-PAGE and transferred to nitrocellulose using an Owl semidry transfer apparatus. The blot was blocked in 10% milk in 1× phosphate-buffered saline (PBS) with 0.1% Tween (PBS-T) and probed overnight at 4°C with an antibody raised against the IE2 C terminus (MAB8140; Millipore Sigma) diluted 1:1,000 in 2% milk–PBST. For the actin loading control, the blot was probed for 1 h with rabbit anti-β-actin antibody diluted at 1:2,000 in 2% milk in PBS-T. Afterwards, the blot was washed 3× for 7 min with PBS-T and incubated for 1 h at RT with a secondary horseradish peroxidase (HRP)-conjugated goat anti-mouse or goat anti-rabbit antibody diluted at 1:40,000 in 2% milk in PBS-T. The blot was subsequently washed 3× for 7 min with PBS-T and imaged on an analytikjena UVP ChemStudio with SuperSignal West Femto maximum sensitivity substrate (34094; Thermo Scientific).

### RNA-Seq.

Total RNA was isolated from DMSO- or dTAG-treated HFF infected with HCMV Towne IE2-FKBP virus for 96 h with TRIzol according to the manufacturer’s protocol. DMSO treatment was carried out for the last 6 h of infection and dTAG treatment for the last 6 or 12 h of infection. All conditions were assayed in biological triplicate. Isolated total RNA pellets from the TRIzol extraction were resuspended in a DNase digestion buffer containing 2 U Turbo DNase (AM2238; Invitrogen), its reaction buffer, and 20 U SUPERase-in RNase inhibitor and incubated at 37°C for 30 min. The DNA digestion reactions were then quenched with the addition of 300 μL TRIzol, and RNA was subjected to column purification using a Zymo Direct-zol RNA mini-prep kit (R0250; Zymo) according to the manufacturer’s instructions. RNA concentrations were determined by NanoDrop analysis, and samples were submitted for quality assessment at the University of Iowa Institute of Human Genetics. Bioanalysis on an Agilent Bioanalyzer 2100 confirmed all samples to have RIN scores of >9, and Lunatic spectrophotometer measurements suggested no significant contamination of the samples by DNA or other organics. Total RNA-Seq libraries were prepared by the University of Iowa Institute of Human Genetics using a TruSeq-stranded total RNA library prep gold kit (200020598; Illumina) and sequenced on an Illumina Nova-Seq 6000 with 50-bp paired-end reads.

### Standard ChIP-Seq.

A T-75 flask of HFF grown to contact inhibition were infected with HCMV TB40/E IE2-GFP at an MOI of 3 for 48 h and then cross-linked with addition of 1% PFA to the medium for 10 min. Crosslinking was quenched by the addition of Tris, pH 7.8, to the medium at 1.33 M. Cells were scraped from the plate surface and pelleted by centrifugation at 1,200 × *g* in a refrigerated centrifuge. Pellets were decanted of supernatant and resuspended in ice-cold 1× PBS supplemented with 0.1%, vol/vol, isopropanol-saturated phenylmethylsulfonyl fluoride (PMSF) and then pelleted once more. Cell pellets were stored at −80°C. Frozen pellets were resuspended in 600 μL sonication buffer (20 mM Tris, pH 7.6, 150 mM sodium chloride, 1 mM EDTA, and 0.2%, wt/vol, Sarkosyl, completed with 0.1%, vol/vol, isopropanol-saturated PMSF). Samples were sonicated in a chilled QSonica sonicator water bath (30 s on, 30 s off, 30% amplitude) for 10 cycles (IE2 ChIP Rep 1, Pol II ChIP) or 20 cycles (IE2 ChIP Rep 2). Afterwards, 60 μL 10% Triton X-100 was added to each and mixed. The samples were then spun at 13,000 rpm in a refrigerated centrifuge for 15 min, and the supernatant was saved in a DNA Lo-Bind tube (022431005; Eppendorf). Samples were precleared by the addition of 25 μL of a 50% slurry of virgin agarose beads and rotation at 4°C for 30 min. Beads were settled and the supernatant was saved. For IE2 ChIP, 25 μL of a 50% slurry of Chromotek GFP-Trap beads (gta-10; virgin agarose also provided) equilibrated in sonication buffer was added to the samples and incubated with rotation for 3 h at 4°C. For Pol II ChIP, 2.5 μg of an antibody that recognizes the POLR2A N terminus (sc-55492; Santa Cruz) was added to the sample and allowed to incubate for 2 h at 4°C. For the Pol II IP, after 2 h, 60 μL of a 50% slurry of protein A-conjugated Sepharose beads equilibrated in sonication buffer was added to the sample and allowed to incubate with rotation for an additional hour. Afterwards, beads were settled for all samples and subjected to washing once in 200 μL wash buffer A (20 mM Tris, pH 7.6, 150 mM NaCl, 1%, vol/vol, Triton X-100, 0.1%, wt/vol, sodium deoxycholate, 0.1%, wt/vol, sodium dodecyl sulfate, and 1 mM EDTA), once in 200 μL wash buffer B (20 mM Tris, pH 7.6, 500 mM NaCl, 1%, vol/vol, Triton X-100, 0.1%, wt/vol, sodium deoxycholate, 0.1%, wt/vol, sodium dodecyl sulfate, and 1 mM EDTA), once in wash buffer C (20 mM Tris, pH 7.6, 250 mM lithium chloride, 0.5%, vol/vol, Igepal CA-360, 0.5% sodium deoxycholate, 0.1% sodium dodecyl sulfate, and 1 mM EDTA), and twice in rinse buffer (20 mM Tris, 50 mM NaCl, and 1 mM EDTA). All buffers were supplemented prior to use with 0.1%, vol/vol, isopropanol-saturated PMSF. Washes were carried out for 5 min at 4°C with rotation, and beads were settled by brief centrifugation at 2,000 rpm in a deli centrifuge. After washing, samples were eluted twice with 50 μL elution buffer (20 mM Tris, pH 7.6, 1% sodium dodecyl sulfate, and 1 mM EDTA) at 65°C for 5 min. Eluates were combined and treated with 40 μg RNase A and incubated for 30 min at 37°C. Afterwards, samples were combined with 2 μL proteinase K and incubated for 2 h at 65°C for protein digestion and decross-linking. DNA was then purified on a MinElute column (28004; Qiagen), and DNA concentrations were measured by Qubit HS dsDNA assay (Q32854; Thermo Scientific). Libraries were prepared using a KAPA HyperPrep kit according to the manufacturer’s instructions. Illumina adapters utilized are listed in Data Set S1, nucleic acid reagents. PCR-amplified libraries were subjected to bioanalysis prior to combination in equimolar ratio and size selection (150 to 600 bp) with a Blue Pippen instrument (BDF2010 cassette). Libraries were sequenced on an Illumina NovaSeq-6000 with 50-bp paired-end reads.

### DFF ChIP.

DFF-ChIP libraries were prepared from native isolated nuclei and cross-linked nuclei. Protocols for each varied at the front end. Native nuclei were isolated as previously described (12). Briefly, contact-inhibited HFF infected for 48 h with HCMV TB40/E IE2-GFP were decanted of media, quickly washed in ice-cold PBS, and then immediately lysed in 5 mL of a lysis buffer containing 20 mM HEPES, pH 7.8. 1% Igepal CA-360, 1 mM EDTA, 0.1% isopropanol-saturated PMSF, 1 mM spermine, 1 mM spermidine, 1 mM dithiothreitol (DTT), 0.004 U/μL SUPERase-In, and 320 mM sucrose and supplemented with a cOmplete protease-inhibitor cocktail tablet. Lysates were gently layered atop a 10-mL sucrose cushion containing 20 mM HEPES, pH 7.8, 0.1% Igepal CA-360, 0.1 mM EDTA, 0.1% isopropanol-saturated PMSF, 1 mM spermine, 1 mM spermidine, 1 mM DTT, 0.004 U/μL SUPERase-In, and 1 M sucrose and then centrifuged for 5 min at 22,500 × *g* and 4°C in a swinging-bucket rotor. Samples were aspirated of supernatant and nuclear pellets were further processed as indicated below. For DFF-ChIP of cross-linked nuclei, cross-linking and isolation of cell pellets were carried out as for standard ChIP. Native nuclei were immediately resuspended following isolation in 400 μL of a digestion buffer containing 20 mM HEPES, pH 7.6, 100 mM potassium chloride, 5 mM magnesium chloride, and 1 mM DTT, supplemented immediately prior to use with 0.1%, vol/vol, PMSF. The nuclei were homogenized by Dounce homogenization. The sample was digested by the addition of 10 to 12 μg of purified recombinant DFF for 2 h at 37°C. Crosslinked cell pellets were resuspended in 396 μL of a digestion buffer containing 20 mM HEPES, pH 7.6, 100 mM KCl, 5 mM MgCl, 1 mM DTT, and 0.5%, vol/vol, Triton X-100, supplemented immediately prior to use with 0.1%, vol/vol, PMSF. The cross-linked sample was subjected to RNase A treatment with 40 μg RNase A for 30 min at 37°C, followed by digestion with 10 to 12 μg of DFF for 2 h at 37°C. Following DFF digestion of both native and cross-linked nuclei, the samples were transferred to 0.5-mL sonication tubes and sonicated for 1 cycle at 40% amplitude for 20 s in a chilled water bath to encourage the release of digested DNA from nuclei. The samples were then spun in a refrigerated centrifuge for 15 min to pellet insoluble material, and the supernatants were saved. Preclearing was carried out for the cross-linked sample as described for standard ChIP but was omitted for the native samples to limit the number of sample manipulations that may interfere with transient chromatin interactions of IE2 and Pol II. Immunoprecipitation of IE2 and Pol II were carried out as described for standard ChIP, except that beads were equilibrated in digestion buffer. Beads were washed 5× in 200 μL of a wash buffer containing 20 mM Tris, pH 7.6, 150 mM NaCl, 0.1%, vol/vol, Triton X-100, and 1 mM EDTA. Washes were carried out for 5 min at 4°C with rotation. Samples were then eluted and further processed for sequencing as described for standard ChIP.

### PRO-Seq, ChIP-Seq, DFF-ChIP, and RNA-Seq track generation.

PRO-Seq data sets analyzed in this study were previously published in Li et al. (12), where details of library handling, track generation, and spike-in normalization are available. Sequencing data generated for this study were retrieved from the University of Iowa Institute of Human Genetics server using the wget command and processed as follows. ChIP-Seq and DFF-ChIP data sets were submitted to an in-house-developed automated track generation pipeline called DNAfastqtoBigWig (https://github.com/P-TEFb/DNAfastqtoBigWig), which, in the following order, trims Illumina adapter sequences with trim_galore v0.6.0 (https://github.com/FelixKrueger/TrimGalore), aligns trimmed sequences of at least 18 bp in length to a combined hg38 and HCMV genome assembly using bowtie v1.2.3 (http://bowtie-bio.sourceforge.net/index.shtml), deduplicates aligned reads with dedup (https://github.com/P-TEFb/dedup) on the basis of sequenced unique molecular identifiers and yields a bed file, converts the bed file into bedGraph format with bedtools v2.26, and finally converts the bedGraph file to a binary bigwig format with the Kent UCSC program bedGraphToBigWig. Bash and awk scripts were utilized to subset the DFF-ChIP data sets for generation of tracks plotting fragments of a specified size range. RNA-Seq data sets were downloaded and trimmed of Illumina adapters and poly(A) tail sequences with trim_galore and then mapped to a combined hg38 and HCMV Towne (FJ616285.1) with splicing-sensitive aligner hisat2 (http://daehwankimlab.github.io/hisat2/manual/). Alignment files were subsequently converted to bigwigs. Coverage of host and viral transcripts in triplicate data sets were determined using featureCounts (https://rnnh.github.io/bioinfo-notebook/docs/featureCounts.html), the results of which were analyzed for differential expression using DESeq2. Changes in host gene expression and viral gene expression were analyzed separately using DESeq2. The 6-h DMSO treatment was used as the control group for both the 6-h and 12-h dTAG treatment.

### Peak calling.

For standard IE2 ChIP-Seq data sets, MACS2 (42) was utilized for peak calling on the host and viral genomes from alignment bam files with parameters callpeak -t input.bam -f BAMPE –bdg. SEACR (43) was also utilized to detect peaks in 25- to 40-bp IE2-GFP DFF-ChIP data in stringent mode. We developed a novel algorithm for peak calling from IE2 DFF-ChIP data sets, called DFF-ChIP-Seq Peak (DFF-CSP), which considers that enrichment of small 25- to 40-bp fragments in the data sets hones in on sites of direct IE2 occupancy. Pileups of 25- to 40-bp fragment centers for the host and HCMV TB40/E (KF297339.1) genomes were first generated, and an 8-bp scanning window function that positions on regions of maximum fragment center density with a lower threshold of 10 fragment centers/window was executed. The output is a bed file. Source code for DFF-CSP is available on GitHub at the following link: https://github.com/P-TEFb/DFF-CSP. Resulting 8-bp windows, termed center clusters, were not allowed to overlap but could be abutted. The average center position for each center cluster was computed, and sequence ±20 bp from this single-base-pair position was queried for motif enrichment with MEME.

### Motif analyses.

For standard IE2 ChIP-Seq, motif discovery was carried out with sequences extending ±50 bp from the MACS2 peak summits. For motif discovery from IE2 DFF-ChIP, fasta files were generated using bedtools getfasta, which represented sequence ±20 bp from the average of the fragment centers in the 8-bp window. Motif discovery was carried out using MEME with parameters -mod zoops -nmotifs 3 -minw 7/14 -maxw 14 -objfun classic -revcomp -markov_order 0. Matches to consensus motifs discovered by MEME on the hg38 and TB40/E genomes were identified by FIMO with parameters –oc. –verbosity 1 –thresh 1.0E-4 motifs.meme KF297339.1.fa/hg38. For matching IE2 binding sites to IE2-regulated promoters, the distance between the maximum TSS within 200-bp start regions defined from published PRO-Cap data from HFF infected for 72 h with TB40/E and the nearest IE2 binding site was calculated. Transcription start regions and their respective maximum TSS were defined using tsrFinder (55). To analyze conservation of IE2 binding sites, the 14mer consensus motifs ±50 bp were aligned to the indicated HCMV genomic sequences using NCBI BLAST.

### Fragment distribution plots and fragMaps.

Fragment size frequencies for IE2 DFF-ChIP data mapping to the host and viral genomes were separately calculated and analyzed in Microsoft excel. Normalized plots of fragment size distribution were generated. fragMaps are two-dimensional (2D) heatmaps that display DFF-ChIP data as a function of fragment size and position. The source code for fragMap.py can be found at https://github.com/P-TEFb/fragMap. Genomic intervals centering approximately on an IE2 binding site or collection of IE2 binding sites were chosen for fragMap analysis. In some cases, fragment center fragMaps are shown, which are similar except that they depict the density of fragment centers rather than full fragment coverage. For all fragMaps, the black value was set as the maximum signal in the heatmap, and a gamma correction of 0.5 was applied to aid in the distinction between light and dark patterns.

### Determination of distance between IE2 binding sites and maximum TSS of affected promoters.

The nearest IE2 binding site for all 1,461 maximum TSSs from the HFF TB40e 72-hpi PRO-Cap data set was computed using a python script. The script computed absolute distances between all maximum TSSs and the center of 78 IE2 binding sites from the X-linked IE2-GFP-DFF-ChIP data set. The closest IE2 binding site was assigned to a maximum TSS based on the shortest distance between them. The bedtools v2.27.1 coverage program was used to count 5′ ends covering the ±100-bp region centered on the maximum TSSs from the IE2-FKBP PRO-Seq data sets with or without 6-h dTAG and with or without Flavo. The ratio of dTAG to no dTAG 5′ end counts was computed using MS Excel by dividing the counts from IE2 6-h dTAG over no dTAG for all maximum TSSs. The 7 IE2-repressed TSRs analyzed were reported in Li. et al. (12) and have a ratio of PRO-Seq 5′ ends greater than or equal to 2. Distances between the maximum TSS of these promoters and the nearest IE2 binding site was determined manually.

### Purification of MBP-IE2-p86, MBP-IE2-p40, and MBP-IE2-DBD.

All expression vectors were designed in a modified pMAL vector that directs the isopropyl-β-d-thiogalactopyranoside (IPTG)-inducible expression of the construct under the control of a strong P_tac_ promoter. The IE2 constructs are expressed as a fusion to an N-terminal MBP, which is followed by a linker sequence and TEV digestion site. At the C terminus of the construct is a 6× His tag. gBlocks encoding IE2 p86, IE2 p40, and the core DBD were designed and ordered from IDT. Integration of the gBlocks into the pMAL vector was carried out using a Gibson assembly cloning kit (NEB E5510S). Assembled vectors were transformed into DH5α (NEB C2987) and were selected overnight at 37°C on ampicillin agar plates. Colonies were picked up and grown overnight in LB plus Amp at 37°C, and plasmids were isolated by miniprep. Accuracy of vector assembly was confirmed by agarose gel electrophoresis and Sanger sequencing. Vectors were transformed into NEB Express BL21 (NEB C3037I) and selected on ampicillin agar plates overnight at 37°C. Colonies were picked up the following morning and allowed to grow in LB plus Amp for 8 h at 37°C. At this time, bacterial cultures were pelleted and washed in fresh LB plus Amp, and cultures were then resuspended and transferred to 1 liter LB plus Amp in a baffled Fernbach flask. Cultures were grown to an optical density at 600 nm (OD_600_) of approximately 0.6 at 37°C prior to induction with 0.1 mM IPTG, supplementation with 15 μM zinc chloride, and transfer to 18°C for overnight protein expression. The following morning, bacterial cultures were pelleted at 4,500 × *g* at 4°C in a swinging-bucket rotor. Pellets were decanted of media, resuspended, and washed in ice-cold 1× PBS supplemented with 0.1% isopropanol-saturated PMSF. Subsequent purification schemes for MBP IE2 p86 differed compared to those for MBP IE2 p40 and MBP IE2 DBD in that p86 was subjected to tandem affinity purification on amylose resin and Ni-nitrilotriacetic (Ni-NTA) resin, whereas purification of p40 and the DBD only involved purification on Ni-NTA resin. For MBP IE2 p86, the pellet was lysed in 12 mL of a lysis buffer containing 1× PBS, 0.1%, vol/vol, Triton X-100, 5%, vol/vol, glycerol, 1 mM DTT, and 0.1% PMSF and supplemented with a cOmplete EDTA-free protease inhibitor cocktail tablet. The suspension was sonicated at 20% amplitude (15 s on, 45 s off, 8 cycles). The sample was salted up to 500 mM NaCl and subjected to ultracentrifugation at 45,000 rpm for 45 min. Amylose resin was equilibrated in the high-salt lysis buffer was subsequently combined with the supernatant from the high-speed spin and incubated with rotation at 4°C for 2 h. The resin was loaded into a gravity flow column, and after allowing the unbound material to flow through, the resin was washed 2× with 10 mL high-salt wash buffer (1× PBS, 500 mM NaCl, 0.1%, vol/vol, Triton X-100, 5% glycerol, 1 mM DTT, and 0.1%, vol/vol, isopropanol-saturated PMSF) 2× with 10 mL lysis buffer and finally eluted with 10 mL maltose elution buffer (1× PBS, 0.1% Triton X-100, 10%, vol/vol, glycerol, 100 mM maltose, 1 mM DTT, and 0.1 mM isopropanol-saturated PMSF). The eluate was supplemented with 5 mM imidazole, combined with 1 mL Ni-NTA resin equilibrated in elution buffer, and incubated with rotation at 4°C for 1 h. Subsequently, the sample was loaded into a gravity flow column and allowed to flow through. The resin was washed twice with 2× 10 mL of a buffer containing 1× PBS, 0.1%, vol/vol, Triton X-100, 5% glycerol, 20 mM imidazole, and 0.1%, vol/vol, isopropanol-saturated PMSF. Afterwards, bound IE2 p86 was eluted with 8 mL elution buffer containing 1× PBS, 0.1%, vol/vol, Triton X-100, 10% glycerol, 1 mM DTT, 300 mM imidazole, 15 μM zinc chloride, and 0.1%, vol/vol, isopropanol-saturated PMSF and collected in 1-mL fractions. Purity and peak protein-containing fractions were determined by SDS-PAGE and silver staining of purified protein fractions. Peak fractions were combined and subjected to dialysis in 1× PBS, 10% glycerol, 1 mM DTT, and 0.1%, vol/vol, isopropanol-saturated PMSF. Protein concentrations following dialysis were approximated by OD_280_ measurement on a Thermo Fisher NanoDrop. Purification of IE2 p40 and the DBD was carried out on Ni-NTA resin alone and followed the initial steps described for purification of IE2 p86, with the exception that supernatants were first combined with 1 mL equilibrated Ni-NTA resin and then processed as described above.

### *In vitro* transcription.

A CMV promoter-driven *in vitro* transcription template harbored within a pGL3 vector was modified by site-directed mutagenesis to functionally eliminate the *crs* present in the CMV core promoter region. This control template was secondarily modified by PCR to insert the IE2 binding site CGTTTTGGAAAACG 150 bp downstream of the TSS (IE2 EB template). Control and IE2 EB templates were PCR amplified with a biotinylated forward primer, purified using a Thermo Fisher PureLink PCR purification kit (K310001; Thermo Fisher), and subsequently immobilized to Dynabeads M280 Streptavidin (11206D; Invitrogen) per manufacturer recommendations. On each template, preinitiation complexes were assembled through incubation with HeLa nuclear extract for 30 min in 20 mM HEPES, pH 7.8, 62.5 mM potassium chloride, 5 mM magnesium chloride, 0.5 U/μL SUPERase-in, and 1 mM DTT. Transcription was initiated by the addition of a pulse mixture containing 20 mM HEPES, pH 7.8, 60 mM potassium chloride, 5 mM magnesium chloride, 1 mM DTT, 1.5 mM AUG mixture (final concentration of 0.5 mM each nucleotide), and α-[^32^P]CTP for 3 min. The pulse was quenched with the addition of 100 μL high-salt EDTA wash (HSWE; 20 mM HEPES, pH 7.8. 1.6 M potassium chloride, 50 mM EDTA, and 0.02%, vol/vol, Tween 20). The beads were concentrated with a magnet and washed twice more with 5-min incubations with HSWE and then twice with low-salt wash (LSW; 20 mM HEPES, pH 7.8, 60 mM potassium chloride, and 0.02%, vol/vol, Tween 20). The isolated transcription elongation complexes were subjected to a pretermination protocol that removes remnant TTF2 function through a 10-min incubation in 20 mM HEPES, pH 7.8, 60 mM potassium chloride, 5 mM magnesium chloride, 0.5 mM ATP, 1 μM single-stranded oligonucleotide, 1 mM DTT, and 0.2 U/μL SUPERase-in. The pretermination reaction was quenched with the addition of 100 μL low-salt EDTA wash (LSWE; 20 mM HEPES, pH 7.8, 60 mM KCl, 25 mM EDTA, and 0.02%, vol/vol, Tween 20). Beads were washed once more in 200 μL LSWE, twice in 200 μL LSW, and finally resuspended in a sample buffer containing 20 mM HEPES pH 7.8, 60 mM KCl, 5 mM MgCl, and 0.2 U/μL SUPERase-in. A sample representing the unchased material was reserved for analysis. Elongation complexes were aliquoted and incubated with a titration of IE2 p40 (1, 3, and 10 pmol), IE2 DBD (1 and 3 pmol), or IE2 p86 (1 pmol). Amounts are approximate, as quantification of protein levels could not be absolutely determined due to small amounts of Triton present in the purified protein samples. Samples were incubated for 10 min and then subjected to a 20-min chase with the addition of 4 μL of a chase mix containing 20 mM HEPES pH 7.8, 60 mM potassium chloride, 18 mM magnesium chloride (final 3 mM), 1 mM DTT, and 3 mM AUGC mix (final concentration, 0.5 mM each nucleotide). Reactions were quenched with the addition of 200 μL Torula yeast stop solution (100 mM Tris, pH 7.6, 0.2 mg/mL Torula yeast RNA, 20 mM EDTA, 1% Sarkosyl). Nucleic acids were isolated via phenol extraction and ethanol precipitated. Samples were resuspended in 10 μL RNA loading buffer and separated on by 6% urea-TBE PAGE. The gel was stained with ethidium bromide to visualize template DNA, dried, and exposed to a phosphor screen. Signals were detected using a GE Typhoon FLA 7000 instrument, and data were processed using FUJI Multigauge software. To examine the effect of IE2 on transcription in the presence of all factors in extract, IE2 p40 was titrated into aliquoted PIC formation reactions. Reactions were pulsed as described above for 30 s and then chased for 3 min with the addition of CTP to 500 μM. Reactions were stopped and nucleic acids were extracted and analyzed as described above.

### RT-qPCR.

Whole-cell RNA was isolated by using TRIzol reagent (15596026; Invitrogen), and reverse transcription (RT) was performed using SuperScript III reverse transcriptase (18080093; Invitrogen) according to the manufacturer’s instructions. PCR primers are listed in Table S1. Quantitative real-time PCR (qPCR) was quantified on the Applied Biosystems 7500 Fast real-time PCR system using the standard curve method. All RT-qPCR analyses were performed in triplicate infections and triplicate treatment conditions in 12-well plates, and results were normalized to host glyceraldehyde-3-phosphate dehydrogenase (GAPDH) RNA. Power SYBR green PCR master mix (4367659; Thermo Fisher) was used with PCR parameters of 95°C for 10 min, followed by 40 cycles at 95°C for 15 s and 60°C for 60 s.

### EMSA.

Individual probes were combined in equimolar ratio and annealed in 20 mM HEPES, pH 7.8, and 100 mM NaCl by heating to 95°C for 5 min and then gradually cooling the sample to room temperature. The probe and IE2 DBD were each diluted in binding buffer (25 mM HEPES, pH 7.8, 100 mM NaCl, 0.01%, vol/vol, Triton X-100, and 1 mM DTT). A titration of the probe was combined with a fixed amount of IE2 DBD in a total volume of 16 μL and incubated at room temperature for 10 min. Samples were supplemented with 4 μL loading buffer (25 mM HEPES, pH 7.8, 20%, wt/vol, Ficoll) and immediately separated by 4% native-PAGE in 0.5× Tris-glycine buffer. The gel was subsequently silver stained and scanned for analysis.

### Statistics.

Statistics for differential gene expression analysis were generated by DESeq2. For the comparison of distances between IE2-activated, repressed, and nontarget promoter TSSs and the nearest IE2 binding sites, Mann-Whitney tests were performed using GraphPad Prism. Normality tests performed in GraphPad Prism indicated that the distributions of distances were nonnormal. E values relating to motif enrichment and sequence conservation are generated by MEME and NCBI BLAST, respectively. For Pearson’s correlation analysis of ChIP-Seq data sets, the HCMV TB40e genome was divided into 100-bp bins. Bedtools coverage program v2.27.1 was used to compute mapped fragment counts for all 100-bp bins from the IE2-GFP Rep1 and -2 and Pol II HFF ChIP-Seq data sets. The same strategy was utilized for previously published IE2 20-hpi, IE2 72-hpi, and UL84 72-hpi data sets. Counts for all 100-bp bins were normalized by dividing all counts by their sum total. The MS Excel CORREL function was used to generate Pearson’s correlation between all data sets. Colors representing various levels of correlation between data sets are shown in Fig. S2C.

### Data availability.

Raw and processed RNA-Seq, ChIP-Seq, and DFF-ChIP data generated for this study are available at NCBI GEO under accession number GSE193026. Previously published IE2-FKBP depletion PRO-Seq data in TB40/E and Towne and PRO-Seq data utilized for the determination of PSI values are available through NCBI GEO accession number GSE139114. HCMV time course PRO-Seq data and H3K4me3 DFF-ChIP, TBP DFF-ChIP, and UL87 DFF-ChIP data are available at NCBI GEO, accession number GSE185618. Published TB40/E IE2 ChIP-Seq at 20 and 72 hpi and UL84 ChIP-Seq at 72 hpi are available at NCBI GEO, accession number GSE169634.
