# Supplementary material for: Human Cytomegalovirus IE2 Both Activates and Represses Initiation and Modulates Elongation in a Context-Dependent Manner
Source: mBio. 2022 May 17;13(3):e00337-22. doi: 10.1128/mbio.00337-22 (PMC9239164; doi:10.1128/mbio.00337-22)
Supplement: FIG S1 [file mbio.00337-22-s0002.pdf]

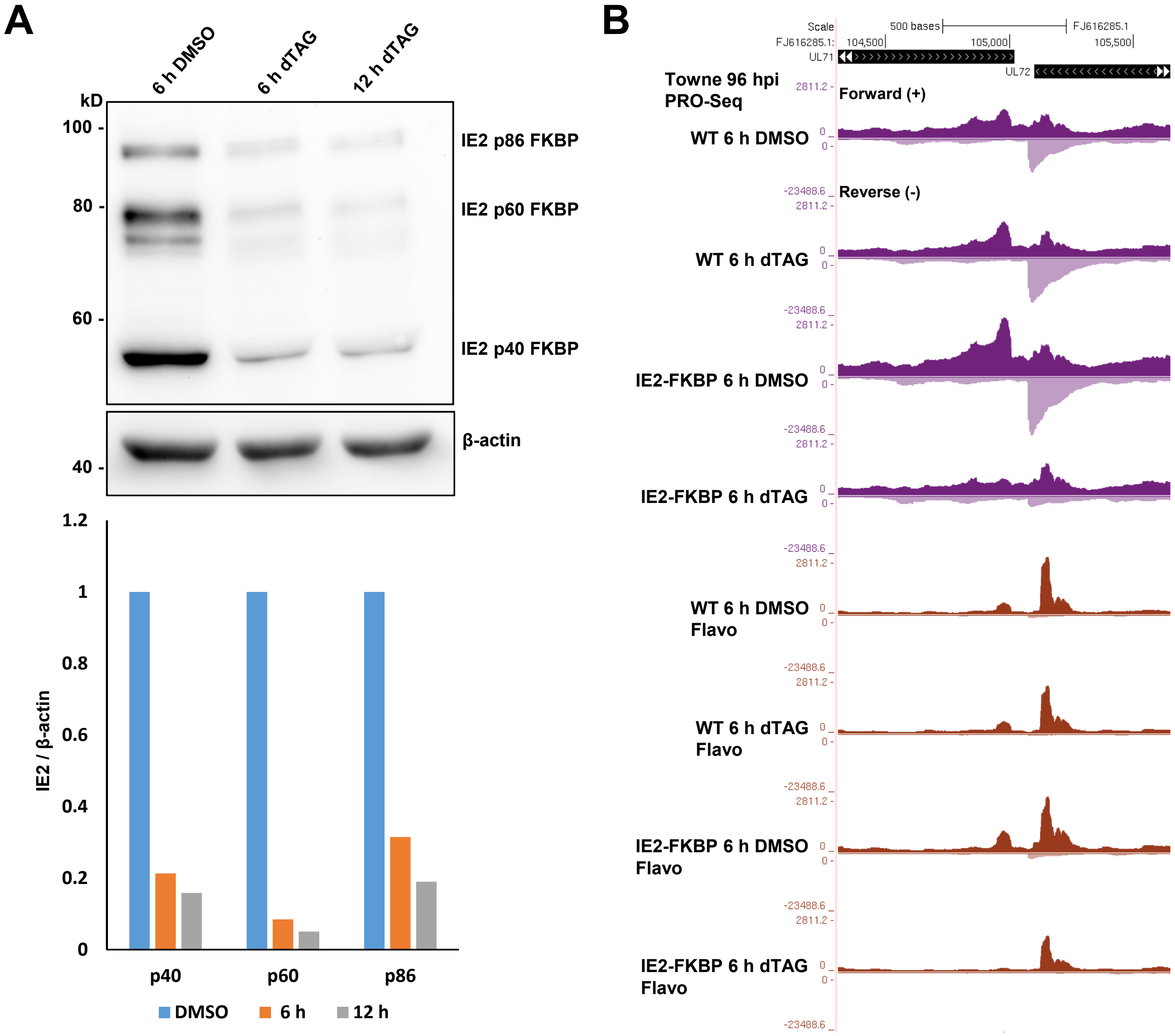
A
6 h DMSO
6 h dTAG
12 h dTAG
kD
100 -
80 -
60 -
40 -
IE2 p86 FKBP
IE2 p60 FKBP
IE2 p40 FKBP
β-actin
IE2 / β-actin
1.2
1
0.8
0.6
0.4
0.2
0
p40
p60
p86
DMSO
6 h
12 h
B
Scale
500 bases
FJ616285.1
FJ616285.1:
104,500
105,000
105,500
UL71
UL72
Towne 96 hpi PRO-Seq
Forward (+)
Reverse (-)
2811.2
0
-23488.6
WT 6 h DMSO
WT 6 h dTAG
IE2-FKBP 6 h DMSO
IE2-FKBP 6 h dTAG
WT 6 h DMSO Flavo
WT 6 h dTAG Flavo
IE2-FKBP 6 h DMSO Flavo
IE2-FKBP 6 h dTAG Flavo

Figure S1. Depletion of Towne IE2-FKBP proteins for RNA-Seq and conservation of the elongation barrier effect in Towne virus. (A) Top: Western blot analysis of IE2 proteins in HFF infected with Towne IE2-FKBP virus for 96 h and treated with DMSO for the last 6 h of infection or dTAG for the last 6 or 12 h of infection. Bottom: Quantification of FKBP-tagged IE2 proteins at the indicated time points relative to the β-actin loading control. (B) Conservation of the elongation barrier effect in Towne virus. DMSO and dTAG treatments of HFF infected for 96 h with wild Towne virus indicate that diminishment of the elongation barrier effect is specific to IE2 depletion and not a result of dTAG treatment.
