# Supplementary material for: Human Cytomegalovirus IE2 Both Activates and Represses Initiation and Modulates Elongation in a Context-Dependent Manner
Source: mBio. 2022 May 17;13(3):e00337-22. doi: 10.1128/mbio.00337-22 (PMC9239164; doi:10.1128/mbio.00337-22)
Supplement: FIG S2 [file mbio.00337-22-s0003.pdf]

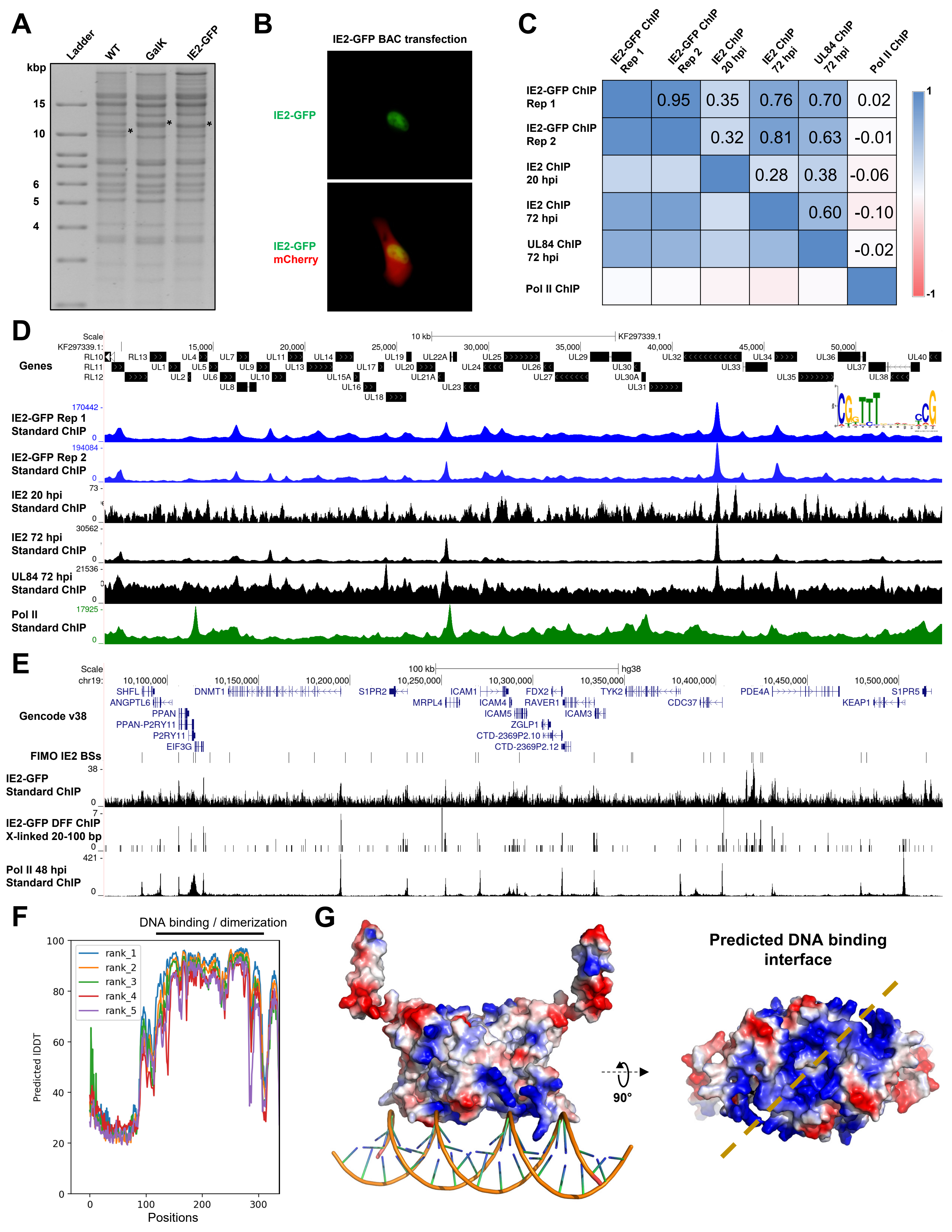
A
Ladder
WT
GalK
IE2-GFP
kbp
15
10
6
5
4
B
IE2-GFP BAC transfection
IE2-GFP
IE2-GFP
mCherry
C
IE2-GFP ChIP Rep 1
IE2-GFP ChIP Rep 2
IE2 ChIP 20 hpi
IE2 ChIP 72 hpi
UL84 ChIP 72 hpi
Pol II ChIP
0.95
0.35
0.76
0.70
0.02
0.32
0.81
0.63
-0.01
0.28
0.38
-0.06
0.60
-0.10
-0.02
1
-1
D
Scale
10 kb
KF297339.1
Genes
IE2-GFP Rep 1 Standard ChIP
IE2-GFP Rep 2 Standard ChIP
IE2 20 hpi Standard ChIP
IE2 72 hpi Standard ChIP
UL84 72 hpi Standard ChIP
Pol II Standard ChIP
170442
194084
73
30562
21536
17925
E
Scale
100 kb
hg38
chr19:
Gencode v38
SHFL
ANGPTL6
PPAN
PPAN-P2RY11
P2RY11
EIF3G
DNMT1
S1PR2
MRPL4
ICAM1
ICAM4
ICAM5
ZGLP1
CTD-2369P2.10
CTD-2369P2.12
FDX2
RAVER1
ICAM3
TYK2
CDC37
PDE4A
KEAP1
S1PR5
FIMO IE2 BSs
IE2-GFP Standard ChIP
IE2-GFP DFF ChIP X-linked 20-100 bp
Pol II 48 hpi Standard ChIP
38
7
421
F
DNA binding / dimerization
rank_1
rank_2
rank_3
rank_4
rank_5
Predicted lDDT
Positions
G
90°
Predicted DNA binding interface

Figure S2. Generation of TB40/E mCherry IE2-GFP, reproducibility of ChIP-Seq data, and further analysis of IE2 DNA binding properties. (A) Restriction fragment polymorphism analysis of wild type TB40/E BAC, a BAC containing a GalK cassette at the IE2 C-terminus, and a BAC containing a GFP coding sequence inserted at the IE2 C-terminus. Asterisks indicate fragments modified to insert the GalK cassette or GFP at the IE2 C-terminus. (B) Live cell fluorescence microscopy image of an HFF transfected with TB40/E mCherry IE2-GFP BAC. IE2-GFP localizes to the nucleus as expected. (C) Pearson's correlation analysis of analyzed ChIP-Seq data sets. (D) Broad genome browser view of IE2-GFP ChIP-Seq, previously published TB40/E 20 and 72 hpi IE2 ChIP-Seq, and previously published 72 hpi UL84 ChIP-Seq. The top MEME motif detected by analysis of sequences from MACS2 peaks called from IE2-GFP ChIP-Seq data is shown in the at the top right of the IE2-GFP ChIp tracks. Peaks of paused Pol II on the HCMV do not correlate with IE2 or UL84 datasets. (E) Broad genome browser view of IE2-GFP ChIP-Seq, 20-100 bp fragments from cross-linked IE2-GFP DFF-ChIP, and Pol II ChIP-Seq on the host genome. These data clearly demonstrate the high quality of Pol II ChIP-Seq data. Peaks in the IE2 ChIP-Seq data modestly correlate with sites of paused Pol II and largely do not overlap with IE2 consensus matches predicted with FIMO. (F) Predicted IDDT values for amino acid positions in IE2 p40 for five ranked models. The approximate location of the DNA binding and dimerization domain is indicated. Higher IDDT values indicate greater confidence in the model. (G) Surface electrostatics for the IE2 p40 dimer (AA 330 - 579) oriented as shown in Figure 2H (left) and rotated 90° to show the predicted DNA binding interface (right).
