# Supplementary material for: Human Cytomegalovirus IE2 Both Activates and Represses Initiation and Modulates Elongation in a Context-Dependent Manner
Source: mBio. 2022 May 17;13(3):e00337-22. doi: 10.1128/mbio.00337-22 (PMC9239164; doi:10.1128/mbio.00337-22)
Supplement: FIG S3 [file mbio.00337-22-s0004.pdf]

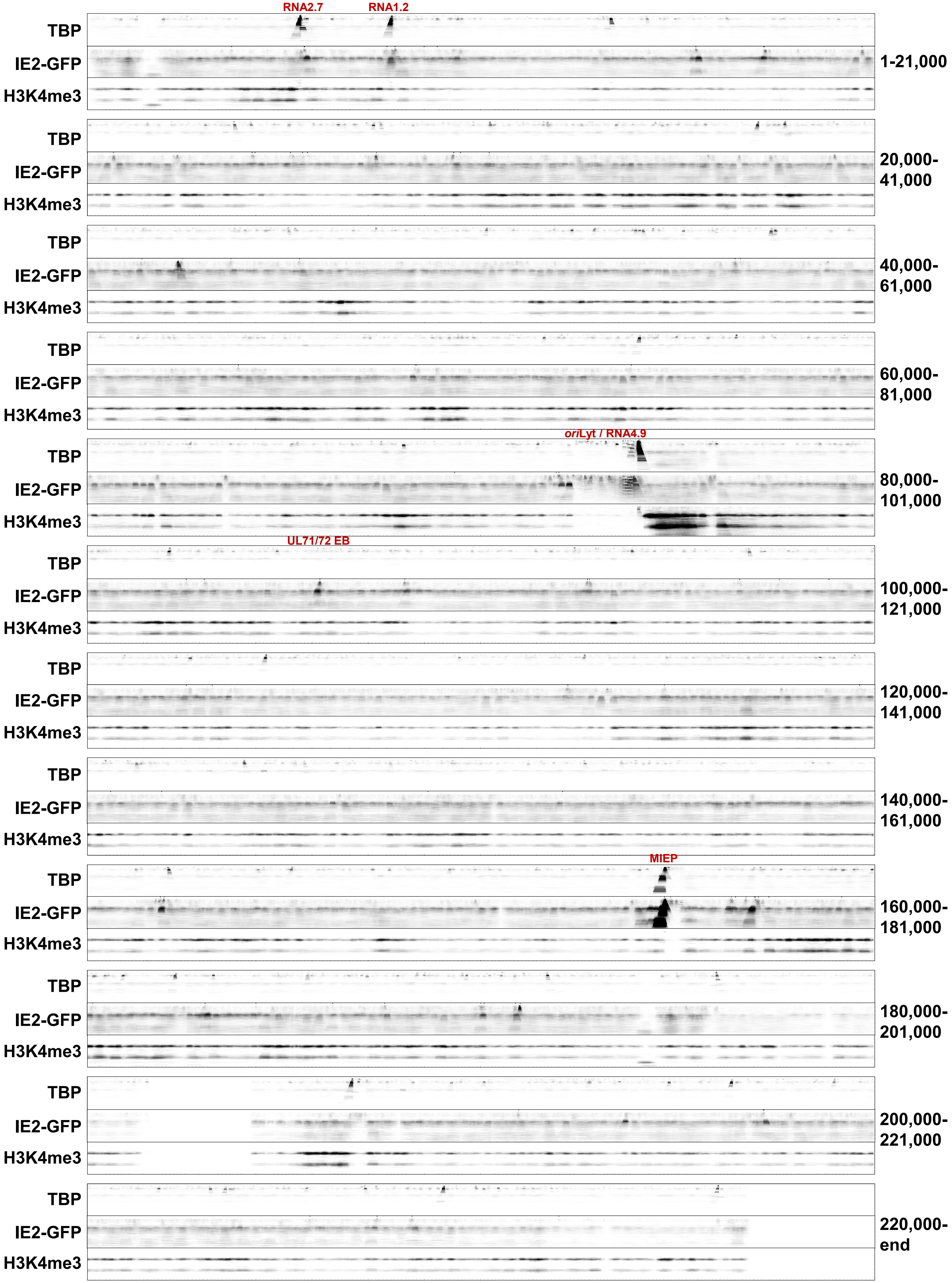

RNA2.7
RNA1.2
TBP
IE2-GFP
H3K4me3
1-21,000
20,000-41,000
40,000-61,000
60,000-81,000
oriLyt / RNA4.9
80,000-101,000
UL71/72 EB
100,000-121,000
120,000-141,000
140,000-161,000
MIEP
160,000-181,000
180,000-201,000
200,000-221,000
220,000-end

Figure S3. HCMV genome-spanning fragMaps for IE2-GFP, H3K4me3, and TBP. 21 kb fragMaps for TBP, IE2-GFP, and H3K4me3 DFF-ChIP at 48 hpi representing the entire HCMV genome are shown. The 78 consensus IE2 binding sites are indicated as small black bars across the top of each IE2-GFP fragMap. Black values for each dataset (TBP, IE2-GFP, or H3K4me3) are set to the same value, enabling comparison of signals across fragMaps. Regions corresponding to RNA1.2, RNA2.7, RNA4.9, and MIEP promoter regions are indicated, as is the site of the UL71/72 elongation barrier. The RNA4.9 promoter region is located at the right side of a broader region designated as *ori*Lyt.
