# Supplementary material for: Human Cytomegalovirus IE2 Both Activates and Represses Initiation and Modulates Elongation in a Context-Dependent Manner
Source: mBio. 2022 May 17;13(3):e00337-22. doi: 10.1128/mbio.00337-22 (PMC9239164; doi:10.1128/mbio.00337-22)
Supplement: FIG S4 [file mbio.00337-22-s0005.pdf]

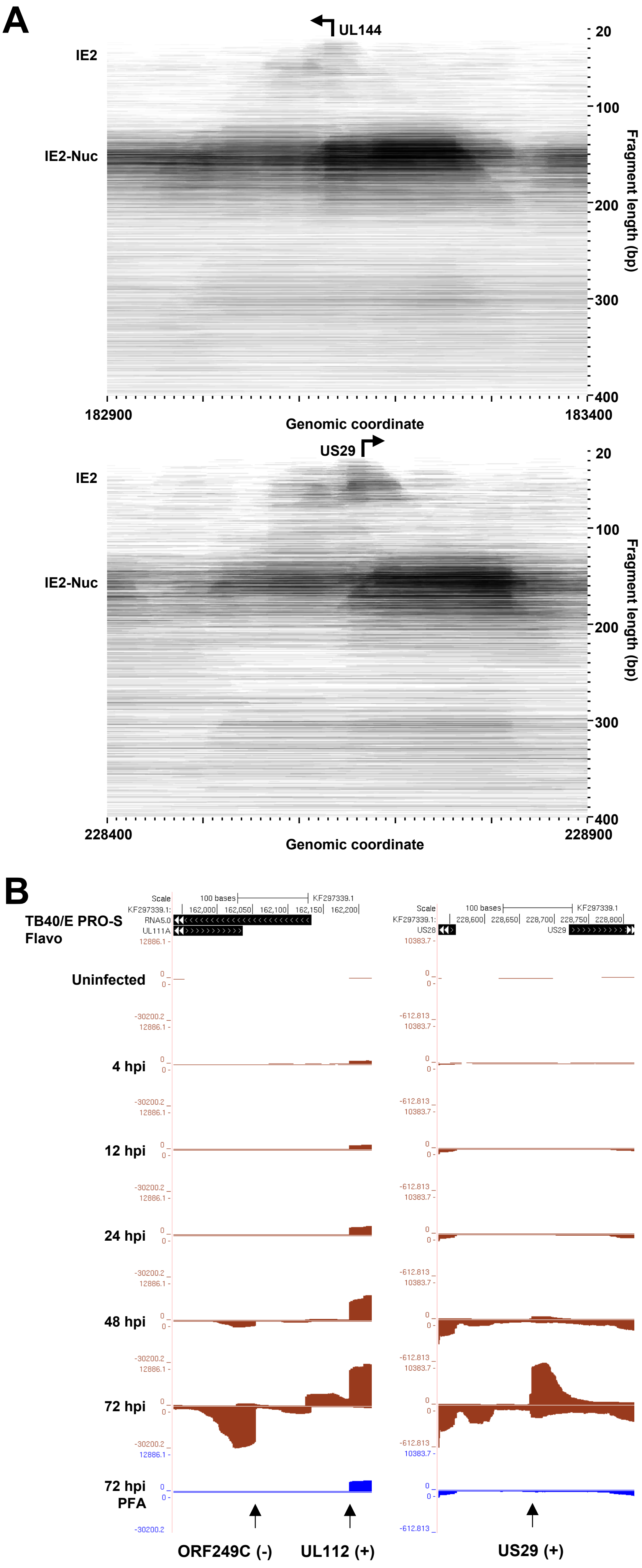
A
UL144
IE2
IE2-Nuc
20
100
200
300
400
Fragment length (bp)
182900
183400
Genomic coordinate
US29
IE2
IE2-Nuc
20
100
200
300
400
Fragment length (bp)
228400
228900
Genomic coordinate
B
TB40/E PRO-S
Flavo
Scale
100 bases
KF297339.1
KF297339.1:
162,000
162,050
162,100
162,150
162,200
RNA5.0
UL111A
228,600
228,650
228,700
228,750
228,800
US28
US29
Uninfected
4 hpi
12 hpi
24 hpi
48 hpi
72 hpi
72 hpi
PFA
12886.1
-30200.2
10383.7
-612.813
ORF249C (-)
UL112 (+)
US29 (+)

Figure S4. Extended analysis of IE2-mediated repression. (A) FragMaps for the UL144 (top) and US29 (bottom) promoter regions. Fragments corresponding to bound IE2 and potentially associated adjacent nucleosomes are indicated, as are the major TSSs. (B) PRO-Seq datasets for an HCMV time course showing a strong induction of the US29 and ORF249LC promoters at 48 hpi, post-replication onset, compared to the UL112 promoter, which is activated early in infection.
