# Supplementary material for: Human Cytomegalovirus IE2 Both Activates and Represses Initiation and Modulates Elongation in a Context-Dependent Manner
Source: mBio. 2022 May 17;13(3):e00337-22. doi: 10.1128/mbio.00337-22 (PMC9239164; doi:10.1128/mbio.00337-22)
Supplement: FIG S5 [file mbio.00337-22-s0006.pdf]

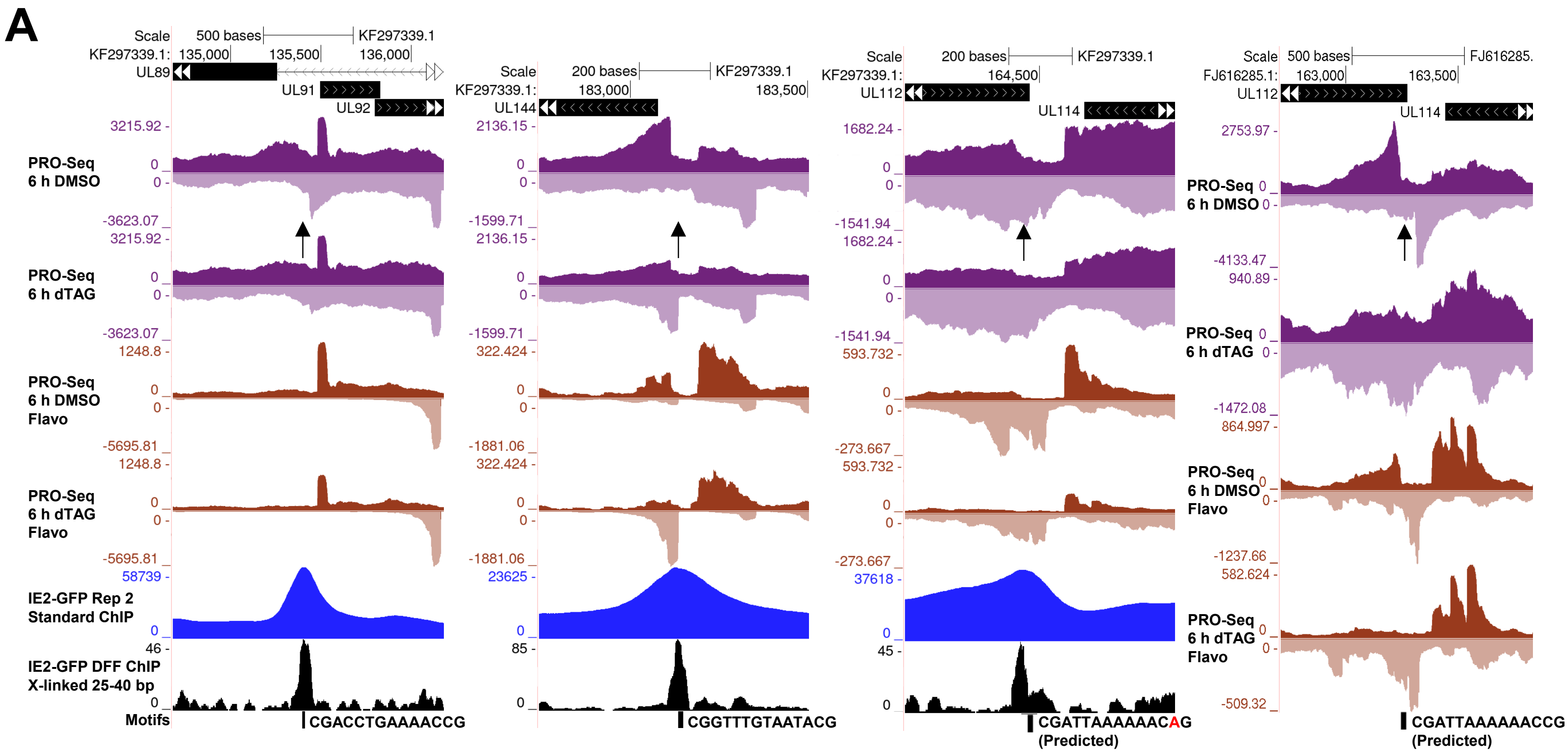

B

Strain	Accession	% Identity	E-value
TB40/e	KF297339.1	100	8.00E-55
Toledo	GU937742.2	100	8.00E-55
VHL-E Merck	KX544841.1	99.12	3.00E-53
Towne	FJ616285.1	99.12	3.00E-53
Merlin	AY446894.1	98.25	4.00E-52

C

WT	CGTTTTGGAAAACG
M1	GCTTTTGGAAAACG
M2	CAGGTTGGAAAACG
M3 *crs*	CGTTTAGTGAACCG

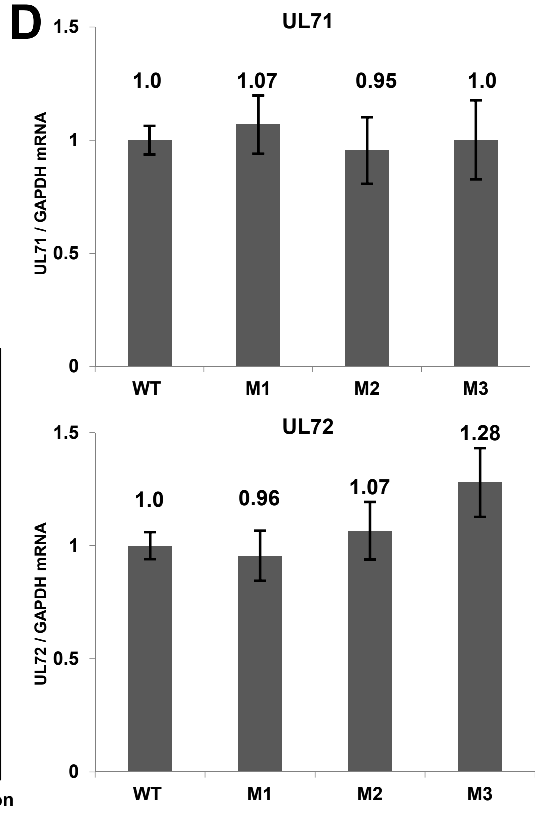

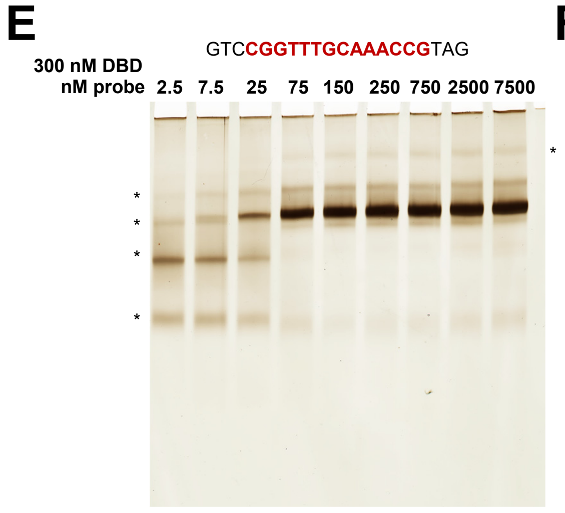

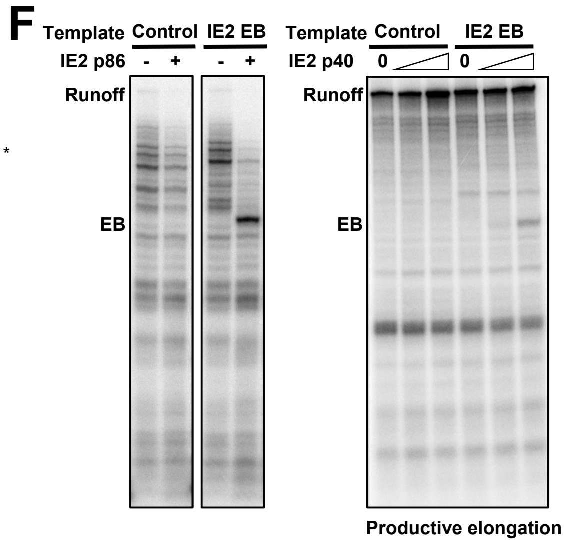

Figure S5. Extended analysis of IE2 elongation barrier function. (A) Examples of IE2 elongation barriers. The center of the barrier is indicated by an arrow, and IE2 occupancy and the corresponding consensus motifs are indicated. (B) Conservation analysis of a 114 bp region centered on the UL71 and UL72 elongation barrier associated IE2 binding site across various laboratory and clinical-like HCMV strains. (C) Designation of the IE2 binding site mutants generated to study the function of the IE2 elongation barrier at the UL71 and UL72 locus. (D) qRT-PCR data at 72 hpi showing unchanged levels of UL71 and UL72 mRNA relative to GAPDH in wild-type and binding site mutant viruses. (E) MBP-IE2 DBD silver stain EMSA showing a laddered pattern of IE2-DBD in the absence of DNA and a shift with the addition of a dsDNA probe representing a perfectly palindromic, 'ideal' IE2 binding site. Asterisks indicate the differentially migrating forms of IE2 observed in the absence and presence of DNA. (F) In vitro transcription assays showing that IE2 p86 blocks Pol II elongation on a template in a reaction context identical to those shown in Fig. 6H and that IE2 p40 partially inhibits Pol II elongation on the template in the presence of all factors in crude nuclear extract.
